# Carbon assimilating fungi from surface ocean to subseafloor revealed by coupled phylogenetic and stable isotope analysis

**DOI:** 10.1038/s41396-021-01169-5

**Published:** 2021-12-11

**Authors:** William D. Orsi, Aurèle Vuillemin, Ömer K. Coskun, Paula Rodriguez, Yanik Oertel, Jutta Niggemann, Volker Mohrholz, Gonzalo V. Gomez-Saez

**Affiliations:** 1 Department of Earth and Environmental Sciences, Paleontology & Geobiology, Ludwig-Maximilians-Universität München, Richard-Wagner-Strasse 10, 80333 Munich, Germany; 2 GeoBio-CenterLMU, Ludwig-Maximilians-Universität München, Richard-Wagner-Strasse 10, 80333 Munich, Germany; 3 Research Group for Marine Geochemistry (ICBM-MPI Bridging Group), Institute for Chemistry and Biology of the Marine Environment (ICBM), University of Oldenburg, Oldenburg, Germany; 4 Leibniz Institute for Baltic Sea Research Warnemünde, Warnemünde, Seestrasse 15, D18119 Rostock, Germany; 5 Alfred Wegener Institute, Helmholtz Centre for Polar and Marine Sciences (AWI), Bremerhaven, Germany

**Keywords:** Microbial ecology, Fungal ecology, Microbiome, Biogeochemistry

## Abstract

Fungi are ubiquitous in the ocean and hypothesized to be important members of marine ecosystems, but their roles in the marine carbon cycle are poorly understood. Here, we use ^13^C DNA stable isotope probing coupled with phylogenetic analyses to investigate carbon assimilation within diverse communities of planktonic and benthic fungi in the Benguela Upwelling System (Namibia). Across the redox stratified water column and in the underlying sediments, assimilation of ^13^C-labeled carbon from diatom extracellular polymeric substances (^13^C-dEPS) by fungi correlated with the expression of fungal genes encoding carbohydrate-active enzymes. Phylogenetic analysis of genes from ^13^C-labeled metagenomes revealed saprotrophic lineages related to the facultative yeast *Malassezia* were the main fungal foragers of pelagic dEPS. In contrast, fungi living in the underlying sulfidic sediments assimilated more ^13^C-labeled carbon from chemosynthetic bacteria compared to dEPS. This coincided with a unique seafloor fungal community and dissolved organic matter composition compared to the water column, and a 100-fold increased fungal abundance within the subseafloor sulfide-nitrate transition zone. The subseafloor fungi feeding on ^13^C-labeled chemolithoautotrophs under anoxic conditions were affiliated with Chytridiomycota and Mucoromycota that encode cellulolytic and proteolytic enzymes, revealing polysaccharide and protein-degrading fungi that can anaerobically decompose chemosynthetic necromass. These subseafloor fungi, therefore, appear to be specialized in organic matter that is produced in the sediments. Our findings reveal that the phylogenetic diversity of fungi across redox stratified marine ecosystems translates into functionally relevant mechanisms helping to structure carbon flow from primary producers in marine microbiomes from the surface ocean to the subseafloor.

## Introduction

Photosynthetic production of marine organic matter and subsequent degradation by heterotrophic microbes regulate global nutrient cycles and the oxidation state of the ocean [1]. The ocean ecosystem is responsible for approximately half of global net primary production, which is carried out by marine phytoplankton including diatoms [2]. Heterotrophic bacteria and archaea help control the flux of carbon, the “biological pump”, which exports biologically produced organic matter in the surface ocean to the deep sea and seafloor [3, 4]. The efficiency of the biological pump exerts a strong influence on Earth’s climate by controlling carbon export to the deep sea [5–7]. The link between heterotrophic microbial activity by bacteria and archaea and the marine carbon cycle is well known [4, 8, 9]. In contrast, heterotrophic fungi have been detected all over the world’s oceans in nearly every marine habitat studied to date, but compared to bacteria and archaea their role in the marine carbon cycle is poorly understood [10, 11].

Fungi are ubiquitous in the ocean [12] and have been isolated from marine habitats including mangrove leaf litter, seagrass, seaweed, subseafloor sediments, and hydrothermal vents [13–17]. Mycoplankton are fungi that live in seawater that tend to correlate with phytoplankton [18–20] and are hypothesized to be an important component for carbon cycling and within marine food webs [10, 11]. Notably, mycoplankton were recently found to dominate total biomass on marine snow [21]. Therefore, we hypothesized that adding dEPS in particulate form to marine samples would enrich fungi that attach to and assimilate carbon from the dEPS particles.

To this end, we used ^13^C DNA stable isotope probing (SIP) to track fungal carbon assimilation of particulate ^13^C-labeled diatom extracellular polymeric substances (^13^C-dEPS) in the water column and sediments underlying the Benguela Upwelling System (BUS) of Namibia. We used ^13^C-dEPS as a tracer for fungal marine organic matter assimilation because diatom derived polysaccharides are major contributors to the marine carbon cycle [22, 23], fungi can dominate total biomass on marine snow [21], and marine fungi are often associated with diatom blooms [18, 19, 24]. The BUS is one of the most productive ecosystems of the world’s oceans [25] and represents a nutrient-rich habitat where the saprotrophic traits of fungi are hypothesized to be important [10–12]. We targeted carbon cycling by two groups of marine fungi, those living as plankton in the seawater (“mycoplankton”) and those living in sediments (“mycobenthos”). We combined the SIP approach with community gene expression (metatranscriptomics), metagenomic sequencing of ^13^C-enriched DNA, quantitative PCR (qPCR), and internal transcribed spacer (ITS) sequencing of the mycoplankton and mycobenthos communities. Microbial analyses were complemented by molecular characterization of the dissolved organic matter (DOM) via Fourier transform ion cyclotron resonance mass spectrometry (FT-ICR-MS). Our findings identify carbon assimilation by key marine fungal groups and characterize the mechanisms underlying carbon cycling by the mycoplankton and mycobenthos. Our findings show that marine fungi can play a quantitatively relevant role alongside heterotrophic bacteria and archaea in the cycling of marine organic matter, and identify key fungal groups and their metabolic mechanisms involved.

## Materials and methods

### Sampling of seawater and sediments

**Fig. 1:**
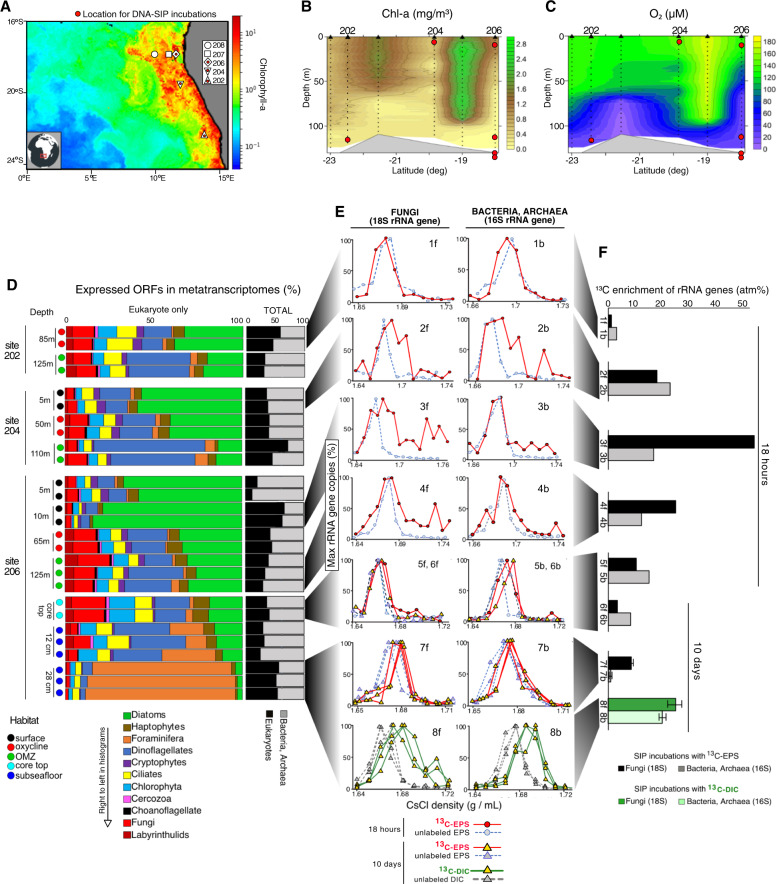
Biogeochemical profiles, gene expression, and microbial carbon assimilation in the BUS. **A** Satellite data of average chlorophyll-a concentrations in the BUS averaged over 7 days period for the EreBUS oceanographic cruise. Sample sites are indicated with symbols, and depth profiles of latitudinal gradients in (**B**) chlorophyll-a and (**C**) dissolved oxygen are shown (together with depths and sites for the SIP incubations). **D** Relative abundance of expressed ORFs in metatranscriptomes of bacteria and archaea versus Eukaryotes (black and gray right-hand plot), as well as the relative abundance of eukaryote groups within the metatranscriptomes (colored left-hand plot). **E** DNA-SIP density gradient qPCR results of 18S rRNA genes with fungi primers (left column) and 16S rRNA genes from bacteria and archaea (right column) (solid lines: ^13^C-labeled, dotted lines: unlabeled controls) from selected sites and depths (shaded gray connector lines). qPCR results from ^13^C-dEPS incubations are in solid red lines, ^13^C-bicarbonate in solid green lines. **F** EAF values for fungal 18 rRNA genes (dark filled histograms) and prokaryotic 16S rRNA genes (light-filled histograms) calculated from the DNA-SIP qPCR results in panel (**E**). The sediment samples derive from three replicate SIP incubations from the same sediment core, and the water column samples are from individual samples (no replicates).

During the *R/V Meteor* oceanographic research cruise ‘EreBUS’’ (2–10 July 2018) seawater was collected at five sites along the Namibian continental shelf (Fig. 1A), sampling each site at two to three depths ranging from 5 m to 125 m (Fig. 1D). Seawater samples and hydrographic profiles were performed using a conductivity-temperature-depth (CTD) probe SBE 911 + (Seabird Electronics, USA), equipped with a rosette sampler consisting of 21 free-flow sample bottles (10 L). Temperature, conductivity, and dissolved oxygen concentration in the water column were measured with Seabird Electronics sensors SBE 3 (temperature), SBE 4 (conductivity), and Clark-type sensor SBE 43 (oxygen concentration). The values of the oxygen sensors were calibrated using Winkler determinations. The fluorescence of Chlorophyll a (Chl a) was measured using a WET Labs FLNTURTD fluorometer. The Chl-a fluorescence was converted into a Chl-a concentration using the calibration data of the manufacturer and shows a qualitative picture of the Chl-a distribution. The sampling of CTD profiles started right below the surface and cover the entire water column to about three meters above the bottom. The vertical speed was 0.5 m/s in the upper 200 m and 1 m/s below. The sampling rate of the CTD probe was 24 Hz. During the processing, outliers were identified before averaging the data to profiles of 0.25 m vertical resolution. The stability of the digiquarz of the CTD was been checked by an external frequency source and no drift was observed. Data along the coastal transect between 23 and 18°S were gridded with the Kriging method and plotted using Surfer software.

Prior to being filled, 1 L borosilicate glass bottles (DURAN) were rinsed three times with the Niskin water before taking the sample. After rinsing, the 1 L glass bottles were filled to the top with seawater, capped with plastic screw caps leaving no air in the headspace. Bottles were fitted with O_2_ sensor spots for constant measurement of dissolved O_2_ throughout the SIP incubations via a non-invasive fiberoptic method as described previously [26]. The first 30 cm of sediment were sampled from a water depth of 125 m from the Namibian continental shelf at Site 206 (18.0 S, 11.3 E) on July 10th, 2018 as described previously [27]. In brief, sediments were sampled with a multi-corer and moved immediately to a 4 °C cold room. Inside the cold room, the core was sampled every 2 cm into nuclease-free 50 mL falcon tubes that were frozen immediately at −20 °C.

### Biogeochemical profiles and DOM analysis

Pore water for measurements of dissolved sulfide and nitrate were extracted at 10 °C from the core and measured as previously described [28]. All water samples for DOM analysis (2 L each) were extracted onboard as previously described [29] using solid-phase extraction (SPE) on styrene-divinylbenzene polymer filled cartridges (1 g, Agilent Bond Elut PPL, USA). The pore water samples (19.5 ± 2 mL each) were filtered and acidified onboard and preserved at 4 °C in the dark. To improve the comparability of the volume-to-resin ratios during the SPE extractions, acidified ultrapure water of pH 2 was added to the pore water samples up to volumes of 0.2 L and cartridges with half of the resin content were used (0.5 g, Agilent Bond Elut PPL, USA). Dissolved organic carbon (DOC) concentrations were measured using a TOC-VCPH analyzer (Shimadzu), from triplicates of the original filtered and acidified water. SPE-DOC concentrations were determined from methanol extracts that were air-dried and re-dissolved in ultrapure water (pH 2). DOC accuracy was evaluated by comparison against a deep Atlantic seawater reference (Consensus Reference Material project: D. Hansell, University of Miami, USA). An extra step of desalination had to be performed prior to FT-ICR-MS analysis by evaporating the methanolic extracts at 40 °C for 24 h, redissolving with acidified ultrapure water, repeating the SPE extraction (0.5 g, Agilent Bond Elut PPL, USA) and removing salts with acidified ultrapure water four times. The desalination step was performed on both the water column and pore water samples. The mean average extraction efficiencies after the desalination process were 43 ± 4 % of the initial DOC. Mass spectra were acquired using a SolariX FT-ICR-MS with a 15 T magnet (Bruker Daltonics) operating in negative ionization mode with an electrospray ionization (ESI) source (Bruker Apollo II) and needle voltage of −4 kV. Analysis was performed in a 1:1 mixture of methanol and ultrapure water, with concentrations of 2.5 ppm C. Samples were measured using direct injection with a flow rate of 1.8 µL min^−1^. An internal deep-sea DOM reference sample [30] was measured twice per day to ensure reproducibility and quality of the mass spectra. As a control to confirm the quality of the spectra samples were injected in triplicates with and without being exposed to the desalination process. For each FT-ICR-MS run, two hundred transient scans in the broadband mode were acquired corresponding to masses ranging from 95–1000 Da. The detection limit was determined using the relative signal intensity, which enables comparisons between samples [31]. Spectra were internally calibrated using the Bruker Daltonics Data Analysis software, together with a calibration list for marine DOM containing >100 known molecular formulas. The data were processed using Matlab pipelines described previously [32], and molecular formulas with C_1-100_, H_1-250_, O_1-100_, N_0-4_, S_0-2,_ or P_0-1_ were assigned to the detected masses. All samples were analyzed twice and the best analytical replicate, in terms of the number of molecular formulas identified, was selected for data interpretation. A total of 13,407 molecular formulas were identified by FT-ICR-MS. Masses were normalized against the summed mass intensities in the sample and used to calculate intensity-weighted averages of molar ratios (N/C, S/C, H/C, O/C, P/C; Supplementary Fig. S1). To assess the degree of saturation of the molecules, the aromaticity index (AI_mod_) was calculated [33, 34]. Seawater samples were grouped depending on their depth and oxygen concentration (see parameters above) into (a) surface (*n* = 3, FT-ICR-MS formulas identified in average = 2515 ± 327), (b) oxycline (*n* = 7, FT-ICR-MS formulas = 2726 ± 267), and (c) oxygen minimum zone (OMZ) (*n* = 8, FT-ICR-MS formulas = 2709 ± 208). Sediment pore water samples were grouped depending on their depth and redox state (see classification above) into (a) core top (*n* = 2, FT-ICR-MS formulas = 2328 ± 202) and (b) subseafloor (*n* = 4, FT-ICR-MS formulas = 2182 ± 399). The pore water sample at 0 cmbsf was discarded from the DOM dataset due to the insufficient quality of the mass spectra. In function of the degree of saturation and oxidation, we classified all molecular formulas into nine chemically robust definitions [32]: (1) Aromatics O-poor (AI_mod_ > 0.5 and O/C ≤ 0.5), (2) Aromatics O-rich (AI_mod_ > 0.5 and O/C > 0.5), (3) Highly unsaturated O-poor (AI_mod_ < 0.5, H/C < 1.5 and O/C ≤ 0.5), (4) highly unsaturated O-rich (AI_mod_ < 0.5, H/C < 1.5 and O/C > 0.5), (5) Unsaturated O-poor (H/C ≥ 1.5, H/C ≤ 2 and O/C ≤ 0.5), (6) Unsaturated O-rich (H/C ≥ 1.5, H/C ≤ 2 and O/C > 0.5), (7) Unsaturated with *N* (former “peptides”, H/C ≥ 1.5, H/C ≤ 2 and *N* > 0), (8) Saturated O-poor (H/C > 2 and O/C ≤ 0.5), (9) Saturated O-rich (H/C > 2 and O/C > 0.5) (Supplementary Fig. S1). Statistical analyses included Principal Coordinate Analysis (PCoA) based on Bray–Curtis dissimilarities, calculated by using R software vegan package [35] following [36].

### DNA and RNA extractions

Immediately after being sampled with the Niskin Rosette, seawater was filtered onboard the ship onto 0.2 µm filters (Pall Supor-200, Port Washington, NY, USA) using a peristaltic pump and an in line filter holder. Filters were then immediately stored in sterile, DNA/RNA clean 15 mL falcon tubes and frozen at −20 °C. DNA was extracted from the filters using a previously described protocol [37]. The DNA from the sediments was extracted from 2 g wet sediment as described previously [28]. DNA concentrations were quantified using a Qubit 3.0 fluorometer (Thermo Fisher Scientific, Waltham, Massachusetts, USA) with the High Sensitivity kit (Invitrogen, Carlsbad, California, USA). RNA was extracted from 2 g wet sediments as previously described using the FastRNA Pro Soil Direct Kit (MP Biomedicals) [28]. RNA was extracted from filters by adding four mL of RNA lysing solution from the FastRNA Pro Soil Direct Kit (MP Biomedicals) in 15 mL Lysing Matrix E tubes (MP Biomedicals) that contained the frozen filters. Filters were then homogenized for 40 s in the FASTprep 5-G homogenizer (MP Biomedicals) at a setting of 6 m per second, centrifuged for 5 min at 13,000 rpm, and the supernatant removed. A second homogenization was performed, this time using 500 µL RNA Lysing Buffer, spun down, and combined with that resulting from the first homogenization. The protocol from the FastRNA Pro Soil Direct Kit (MP Biomedicals) was then followed with the addition of glycogen at a concentration of 1 µg/mL to the isopropanol precipitation. In order to reduce DNA contamination, all RNA was extracted in an RNA-dedicated HEPA-filtered laminar flow hood. To remove potentially contaminating nucleic acids, all surfaces and pipettors were treated with RNAse-Zap and exposed to UV light for 30 min before and after completing the RNA extractions.

### ITS1 sequencing

The fungal ITS1 region from the extracted DNA was amplified with the primer pair ITS1-F/ITS2 (ITS1f: 5’-CTTGGTCATTTAGAGGAAGTAA-3’, ITS2: 5’-GCTGCGTTCTTCATCGATGC-3’) [38, 39] that also contained Illumina adapters and a unique barcode sequence for sample demultiplexing [26]. We acknowledge that these primers are biased against specific groups of Fungi, including the Mucoromycotina, Malasseziales, and Chytridiomycota [40], and we do not claim to have detected all taxa present in our samples but rather only discuss those detectable with these primers. Three PCR replicates were prepared for each sample with the epMotion 5070 robotic pipetting system (Eppendorf) and purified using with QIAquick Gel Extraction Kit (Qiagen) as described previously [26]. Barcoded ITS1 amplicons were pooled at 1 nM and sequenced as described previously [26] on the MiniSeq platform (Illumina) (Supplementary Table S1). The ITS1 sequencing and OTU clustering protocol has been calibrated using mock communities of fungi, which was determined to capture a realistic picture of fungal OTU richness [26].

For the ITS1 quality control and OTU clustering was performed using USEARCH version 10 [41] as described previously [26], with 97% sequence identity and taxonomy was assigned against the UNITE database release 8 [42] using BLASTn in the QIIME2 classifier [43]. We only considered OTUs to be derived from Fungi from the samples (no contaminants) if they had >10 sequences and were not detected in the negative control. The negative controls were extraction blanks and dust samples from our lab that had ITS1 from fungi sequenced in an earlier study [26]. Fungal OTUs in these contaminant controls are used as a list of common lab contaminants that we remove from sample datasets by clustering them together and removing OTUs detected in the contaminants and the environmental samples [26]. Less than 5% of the OTUs were detected in the contaminant samples and removed. As an additional quality control step for accurate annotations, we performed BLASTn searches against NCBI-nr of selected high abundance ITS1 OTUs that were annotated only in the UNITE database as Fungi but not to any taxonomic levels below. These hits appear with the UNITE annotation “Fungi; unclassified”. Cross-checking these annotations against NCBI-nr revealed that many high abundance ITS1 OTUs that were annotated as “Fungi; unclassified” were actually Rhizaria (protists) and invertebrate animals (Cnidaria). Therefore, the ITS1-F/ITS2 primers also amplify non-fungal sequences and to be as conservative as possible we only considered OTUs that had a classification in the UNITE database to the Class level since when we double-checked these with BLASTn against NCBI-nr they all returned hits to fungal taxa. OTUs passing the quality control steps had their relative abundance normalized by the percentage of mapped reads as a proportion of total sequencing depth per sample.

### qPCR

qPCR quantification of the 16S rRNA genes was performed using the primer pair 515 F/806 R with a qPCR protocol that has been previously described [26, 44]. Three technical replicates were prepared with the epMotion 5070 robotic pipetting system (Eppendorf) in white 96-well plates (BioRad) and qPCR was conducted in a CFX Connect real-time PCR system (Bio-Rad, Hercules, CA, USA) [26, 44]. qPCR to quantify 18S rRNA genes from fungi used the fungal specific primers FR1 (5’-AICCATTCAATCGGTAIT-3’) & FF390 (5’-CGATAACGAACGAGACCT-3’), which have high specificity for soil fungi [45]. In one marine sample from Helgoland (Germany), there was co-amplification of non-target eukaryotes observed, but two other marine samples showed more specific fungal amplification [46]. The FR1/FF390 qPCR reactions 20 µl in volume that contained 4 µl of the DNA template,10.4 µl SsoAdvanced SYBR green PCR buffer (Bio-Rad, Hercules, CA, USA), 6.8 µl of nuclease-free (DEPC-treated) water, and 0.4 µl of each primer (10 mM). The FR1/FF390 qPCR protocol consisted of three steps: (1) denaturation for 95 °C for 15 s, (2) annealing at 50 °C for 30 s, and (3) elongation at 72 °C for 90 s. qPCR was carried out for 40 cycles. No template controls were run for every qPCR reaction to monitor contamination in the qPCR reagents, which had consistently cycle threshold values >35.

The standard curves of 16S and 18S rRNA gene qPCR consisted of a 10-fold dilution series (spanning 10^7^–10^1^ gene copies) of either 16S or 18S rRNA genes that were PCR amplified and gel extracted from the sample using the same primers. The amplified standard was quantified with a Qubit 3.0 fluorometer (as described above), prior to the creation of the dilution series. Dilution series were prepared with the epMotion 5070 robotic pipetting system (Eppendorf) to ensure consistency between qPCR efficiency and, therefore, more precise comparisons of gene quantities. The reaction efficiencies in all qPCR assays were between 90% and 110%, with an *R*^2^ > 0.9. For sediment samples, gene copies were normalized to the wet weight of sediment, and for water column samples, gene copies were normalized to mL of water filtered.

### Metatranscriptomics

We sequenced and analyzed metatranscriptomes from 27 samples at three sites spanning the surface, oxycline, OMZ, core top, and subseafloor habitats (Fig. 1D, Supplementary Table S2). Metatranscriptome libraries were prepped as described previously [28] using the Trio RNA-Seq kit protocol (NuGEN Technologies) and sequenced using 2 × 150 bp paired-end sequencing kits on the MiniSeq (Illumina). Sequence quality control, parameters of *de novo* assemblies, and open reading frame (ORF) searches were conducted as previously described [27]. Quality control and *de novo* assembly were done using CLC Genomics Workbench 9.5.4 (https://www.qiagenbioinformatics.com/) with the following parameters: bubble size = 50, word size = 20, and a minimum contig length = 300 nucleotides. Reads were then mapped to the contigs using the parameters: insertion penalty = 3, minimum alignment length = 50% of read length, deletion penalty = 3, mismatch penalty = 3, minimum percent identity = 95%. Eukaryotic ORFs predictions were made using eukaryotic code for translations with TransDecoder v5.5.0 [47].

### Gene identification

ORFs in metatranscriptomes were queried against the MetaProt database as previously described [27] using BLASTp with DIAMOND version 0.9.24 [48]. MetaProt is a custom aggregated database consisting of all predicted protein sequences from NCBI-nr, JGI, RefSeq, SEED, and the eukaryotic transcriptome project MMETS [49]. Queried ORFs having BLASTp hits in the MetaProt database were only considered if they met the following criteria: bit score >50, amino acid similarity >30, alignment length > 50. ORFs were annotated as fungal-derived if they had a predicted protein from a fungal transcriptome or genome as the best BLASTp hit using these stringency criteria. This criterion is sufficient for taxonomic assignment of ORFs from metatranscriptomes to major eukaryotic groups (e.g., Class level and above) [27] and was used to assign ORFs to major eukaryotic groups (Fig. 1D). Fungal ORFs were, furthermore, queried against the Cluster of Eukaryotic Orthologous Genes (KOG) [50] using the same criteria to assign fungal ORFs to broad functional categories.

ORFs were normalized based on the number of (presence/absence) uniquely expressed ORFs because as we observed previously with this particular metatranscriptome library prep [27] this is much more consistent between replicates compared to read mapping (RPKM) values. Since all metatranscriptomes were prepared with the same amplification method, the systematic bias in the amplification step should be the same between all samples and allow for comparisons of relative changes in the transcriptional profile between samples. However, we acknowledge that the absence of ORFs in some samples (Supplementary Table S2) might be caused by insufficient sequencing depth or increased relative abundance of other transcripts stemming from the amplification bias.

The selected ORFs with similarity to functional genes were aligned together with their top BLASTp hits using MUSCLE [51]. Phylogenetic analyses were performed with PhyML [52] in SeaView version 4.7 [53] using BLOSUM62 for the evolutionary model. Statistical support for internal tree nodes was evaluated using 100 bootstrap replicates. Putative carbohydrate-active enzymes (CAZymes) encoded within transcripts were identified by BLASTp searches of ORFs against the CAZyme database [54]. Potentially contaminating ORFs were identified via metatranscriptomes from extraction blanks and the microbial diversity in dust samples collected from the same lab where the RNA extractions and libraries were prepared [26]. This revealed a list of likely contaminant organisms, and ORFs annotated as contaminating organisms in this list were removed. All metatranscriptomes had <10% ORFs from likely laboratory contamination.

In order to find fungal 18S rRNA transcripts within the metatranscriptomes, a portion of the SqueezeMeta [55] metagenomic analysis pipeline was used for rRNAs prediction using co-assembly mode. In short, Trimmomatic [56] was used for adapter removing, trimming, and quality filtering by setting the parameters: sliding window = 10:15, leading = 8, minimum length = 150, trailing = 8. Contigs were assembled using a Megahit assembler [57] using the minimum length of 200 nucleotides. Barnnap [58] embedded in SqueezeMeta was deployed to search the rRNA sequences. Preliminary assignments of taxonomy were made using QIIME 1.9.1 [59] with BLASTn, searching against the SILVA database version 132 [60]. Putative fungal 18S rRNA sequences in the metatranscriptomes were double-checked for annotation accuracy by BLASTn searches against the NCBI-nr database followed by alignment against their closest database hits via multiple sequence alignment using MUSCLE [61]. Alignments were manually curated for accuracy in SeaView [53], followed by phylogenetic analysis with PhyML with 100 bootstrap replications [52].

### Setup of ^13^C DNA-SIP incubations

The ^13^C-dEPS was produced from the diatom *Chaetoceros socialis* [62], which was chosen because it is an ecologically relevant phytoplankton species and has a wide geographic distribution [28]. *C. socialis* cultures were grown and isotopically labeled as previously described using ^13^C-sodium bicarbonate as a carbon source [28]. To produce the unlabeled dEPS material for the controls, *C. socialis* cultures were also grown with no ^13^C label added [28]. The ^13^C-labeled (and unlabeled) dEPS was concentrated as particulate organic matter (POM) and used as an inoculum for the SIP experiments. GC-IRMS determined that the atom percent ^13^C enrichment of the labeled dEPS was >50% [28]. Prior to the cruise, the *C. socialis* dEPS was treated twice with a DNAse enzyme (Turbo DNAse, Life Sciences), increasing the incubation times to 1 h, in order to remove ^13^C-labeled DNA from *C. socialis* that may otherwise have accumulated in the heavy fractions of CsCl gradients after ultracentrifugation.

For seawater ^13^C- SIP incubations, water from the Niskin Rosettes was sampled immediately onboard the ship from three sites (site 202, site 204, site 206) at multiple depths (Fig. 1A, B, C) into 1 L borosilicate glass flasks (DURAN). Bottles received either the unlabeled (control) or ^13^C-labeled dEPS at a final concentration of 0.2 mg L^−1^. For each water column incubation, 1 L of seawater was incubated in capped glass flasks with the added substrate leaving no air in the headspace. Bottles were incubated in the dark at 10 °C for 18 h, with continuous monitoring of O_2_ concentrations with a non-invasive fiber optic method described previously [26]. At the end of the incubation, the seawater was filtered onto 0.2 µm filters (Pall Supor-200, Port Washington, NY, USA) using a peristaltic pump and immediately frozen at −20 °C. DNA was extracted from the filters as described above.

In the core top DNA-SIP incubations, the labeled and unlabeled dEPS was added at a final concentration of 200 µg per g sediment. The total organic carbon content of sediments in this region is between 0.5 and 2.3% [63], and therefore we added the dEPS substrates in an amount that was approximately 1–3% of the in situ concentration of organic matter in the sediments. All core handling and experimental setups were performed in a cold room at 10 °C to reduce the effects of temperature on the in situ activity of the microbial community. For the core top incubations, slurries of 2 g of sediment and 18 mL of hypoxic bottom seawater were added to 20 mL sterile (autoclaved) glass vials. Vials were crimp sealed with sterile (autoclaved) gray rubber butyl stoppers leaving no air in the headspace. Core top dEPS SIP incubations were incubated for 18 h and 10 days, providing two separate time points.

In the 23 cmbsf SIP incubations, 20 g of sediment was added together with either the labeled or unlabeled dEPS was added at a final concentration of 200 µg per g sediment in 20 mL sterile glass flasks. Flasks were crimp sealed using sterilized gray butyl rubber stoppers leaving no air in the headspace. The second set of SIP incubations were set up using subseafloor sediments from 23 cmbsf, whereby sediments were incubated in crimp sealed 20 mL glass flasks together with either ^13^C-bicarbonate or unlabeled bicarbonate (as a control) at a concentration of 2 mM. Flasks received either 2 mM 99% ^13^C-labeled or unlabeled (control) sodium bicarbonate (NaHCO_3_; Sigma-Aldrich, St. Louis, MO, USA). All subseafloor SIP incubations from 23 cmbsf were performed in triplicate for 10 days in the dark at 10 °C. O_2_ was measured non-invasively using a Fibox (PreSens Precision Sensing) as described previously [26]. At the end of the 10-day incubation, flasks were frozen at −20 °C. DNA was extracted from the sediment as described above. Sediment SIP incubations were extended for 10 days (instead of 18 h like the water column) because they were strictly anoxic and we expected rates of microbial activity (and ^13^C-assimilation) to be slower as a result.

### Ultracentrifugation and density gradient fractionation

DNA extracted from the SIP incubations were prepared for density gradient centrifugation as described previously [44]. In brief, ultracentrifugation was performed using a TLN-100 Optima MAX-TL ultracentrifuge (Beckman Colter, Brea, CA, USA) near-vertical rotor at 18 °C for 72 h at 165,000 × *g*. For each ultracentrifugation spin, 50 µL of DNA extracted from the SIP incubations was added to a solution of gradient buffer (0.1 M Tris, 1 mM EDTA, and 0.1 M KCl) and cesium chloride (CsCl) in 3.3 mL polyallomer OptiSeal tubes (Beckman Colter, Brea, CA, USA). The starting density was 1.70 g mL^−1^. Density gradients were fractionated into 15 fractions of 200 µL using a fraction recovery system (Beckman Colter, Brea, CA, USA) connected to a syringe pump, and the density of each fraction was determined using an AR200 digital refractometer (Reichert Analytical Instruments, Depew, NY, USA). The DNA in each density fraction was precipitated overnight at room temperature using 2 volumes of polyethylene glycol and 10 mg mL^−1^ glycogen. DNA was pelleted from the density fractions by centrifuging at 13,000 rpm for 40 min, washed with 70% ethanol, air-dried for 15 min, resuspended with 30 µL nuclease-free (DEPC-treated) water, and quantified using a Qubit as described above.

### Estimating excess atom ^13^C-enrichment fraction (EAF)

qPCR of 16S and 18S rRNA genes were performed on each density fraction from the labeled and unlabeled incubations, using the same qPCR setup and cycling conditions as described above. The observed excess atom ^13^C-enrichment fraction (EAF) was calculated for fungal 18S and prokaryotic 16S rRNA genes according to the equations for calculating EAF values from DNA-SIP experiments provided by Hungate et al. [64]. The EAF value should reflect the proportion of labeled carbon atoms that are assimilated into the genomic DNA (or at least large DNA fragments containing rRNA genes). For example, an EAF value of 0.3 would relate to 30% of carbon atoms within the gene targeted by the PCR primers (e.g., 18S rRNA genes of fungi) are ^13^C labeled [64].

### Metagenomics from ^13^C-labeled density gradient fractions

Metagenomes were prepared from selected density fractions in SIP incubations that showed a clear ^13^C-labeling of fungal 18S rRNA genes. DNA from individual fractions within each of the heavy regions (1–4 individual factions per heavy region) in the ^13^C incubations were pooled and metagenomic (Illumina) libraries prepared as previously described [28]. Fungal (eukaryotic) genomes contain introns that preclude *de novo* assembly of functional genes from metagenomes but expressed mRNA transcripts from fungi (eukaryotes) such as those in the metatranscriptomes are spliced together and do not contain introns and thus provide a more optimal means for functional gene annotations. Therefore, raw Illumina reads from the heavy metagenomes were mapped against the complete set of annotated ORFs from the seawater and sediment metatranscriptomes (see metatranscriptome annotation pipeline above), as well as the MetaProt database (described above), using BLASTx (e value cutoff: 10^−10^). This allowed for the identification of heavy ORFs that had similarities to functional fungi (or Bacteria and Archaea). Putatively fungal heavy ORFs that recruited a relatively high number of reads (>10 reads) (NADH dehydrogenase, cytochrome c oxidases, cellulase family E, trypsin, and carbohydrate-binding module 48) were used to identify fungal sequences that contained ^13^C-label. To confirm their annotation as fungi, ^13^C-labeled ORFs were aligned against their best BLAST hit using MUSCLE [61] in SeaView [53], followed by phylogenetic analysis with PhyML [52] with 100 bootstrap replicates.

## Results

### Biogeochemical profiles

**Fig. 2:**
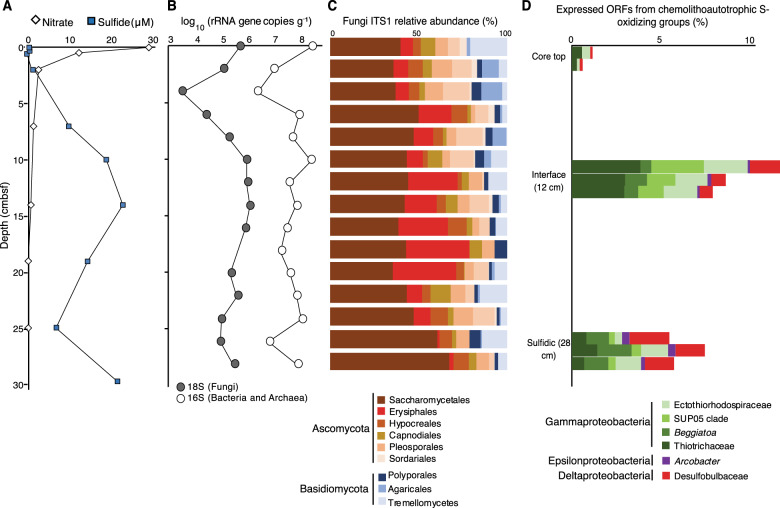
Vertical profiles of pore water chemistry, microbial abundance, fungal diversity, and bacterial gene expression in the sediment core. **A** Vertical profile of pore water nitrate and sulfide. **B** Down-core concentrations of fungal 18S rRNA genes and prokaryotic 16S rRNA genes determined by qPCR. **C** Diversity of fungal ITS1 sequences. **D** Relative expression of ORFs from known groups of chemolithoautotrophic sulfide oxidizing bacteria, the three separate stacked histograms at each of the three depths correspond to replicate metatranscriptomes from the same depth. Note the higher expression from sulfide oxidizing bacterial groups within the sulfide-nitrate transition zone, where fungal abundance increases by two orders of magnitude. cmbsf: centimeters below the seafloor.

Concentrations of Chl-a decreased with distance from shore indicating reduced phytoplankton biomass, whereas higher concentrations of Chl-a and DOC were observed along the coast (Fig. 1A, Supplementary Table S3). Averaged SPE-DOC concentrations (26 ± 2 µM) were within the same range as in the Humboldt Upwelling System [65], with the highest concentrations in the oxycline (75 m) of site 202 (Supplementary Table S3). Consistent with prior studies [25, 66], between 50–95 m an oxycline was observable that exhibited O_2_ concentrations spanning 100–40 µM O_2_ [67]. Below 65–95 m, an oxygen minimum zone (OMZ) core defined as having <60 µM O_2_ [67] was detected along the shelf and at all three sites sampled (Fig. 1C). The sediments exhibited a redox gradient from suboxic (<10 µM O_2_) conditions at the surface sediments to sulfidic (25 µM H_2_S) conditions starting at 5 cmbsf (Fig. 2A). Based on previous classifications for water column O_2_ profiles [67], and sediment pore water H_2_S and NO_3_^-^ profiles [28], the sampled habitats were grouped into five biogeochemical categories: surface ocean (5–10 m water depth: 160–110 µM O_2_), oxycline (50–95 m water depth: 110–40 µM O_2_), OMZ (95–125 m water depth: <60 µM O_2_), core top (suboxic), and subseafloor (12–28 cm below seafloor: sulfidic).

**Fig. 3:**
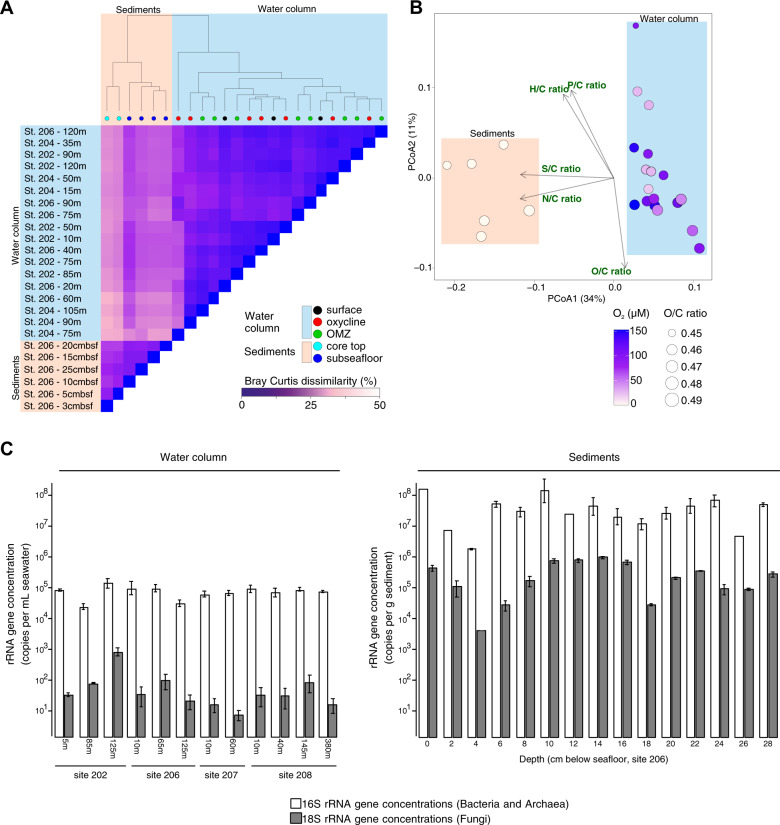
DOM composition and microbial abundance in the seawater and sediments. **A** Molecular dissimilarity analysis (Bray–Curtis) of DOM considering the relative signal intensities of molecular formulas detected via FT-ICR-MS. **B** Principal Coordinate Analysis (PCoA) of DOM based on the Bray–Curtis dissimilarities. The color code represents the concentration of dissolved oxygen and the size of the dots the averaged intensity-weighted normalized O/C ratio in the FT-ICR-MS molecular formulas identified. **C** Histograms show the concentration of fungal 18S rRNA genes and prokaryotic 16S rRNA genes measured via qPCR in the water column and sediment samples. Error bars show standard deviations (*n* = 3 replicates).

Molecular characterization of the DOM composition via FT-ICR-MS showed clear differences between the sediment pore water DOM and the water column DOM in BUS (25 ± 3 dissimilarity, Bray Curtis; Fig. 3A). Most of the variability in the DOM dataset could be explained by the high proportion of compounds containing heteroatoms different than oxygen in the sediments (N, S and P; Figs. 3A, Supplementary S1), while oxygen concentrations did not show a clear effect on the DOM composition in the water column (Fig. 3B). A higher proportion of unsaturated compounds with *N*, such as degraded peptides [68], was detected in the sediment pore water DOM compared to the seawater samples (Supplementary Fig. S1), in accordance with pelagic sediment pore waters from diverse marine environments [69].

### Fungal 18S rRNA gene concentrations

To estimate fungal abundance across the different samples we quantified fungal 18S rRNA genes using qPCR. Fungal 18S rRNA gene concentrations in the water column were relatively low between 10^1^–10^3^ gene copies mL^−1^ (Fig. 3C), and were consistently close to our qPCR detection limit determined from DNA extraction blanks (cycle threshold value = 35). In the underlying sediments, the concentration of fungal 18 S rRNA genes was two to three orders of magnitude higher compared to the water column and ranged from 10^4^–10^6^ gene copies g^−1^ (Fig. 3C). Below the seafloor, concentrations of fungal 18S rRNA genes decrease several orders of magnitude over the top 6 cm (Figs. 2B, 3C). However, between 8 and 18 cm below seafloor (cmbsf) the concentration of fungal 18S and 16S rRNA genes from bacteria and archaea increase by roughly two orders of magnitude within a nitrate-sulfide transition zone (Fig. 2B). In both the seawater and sediments, fungal 18S rRNA genes were on average two orders of magnitude less concentrated compared to the 16S rRNA genes from bacteria and archaea (Fig. 3C).

### Fungal ITS1 diversity

**Fig. 4:**
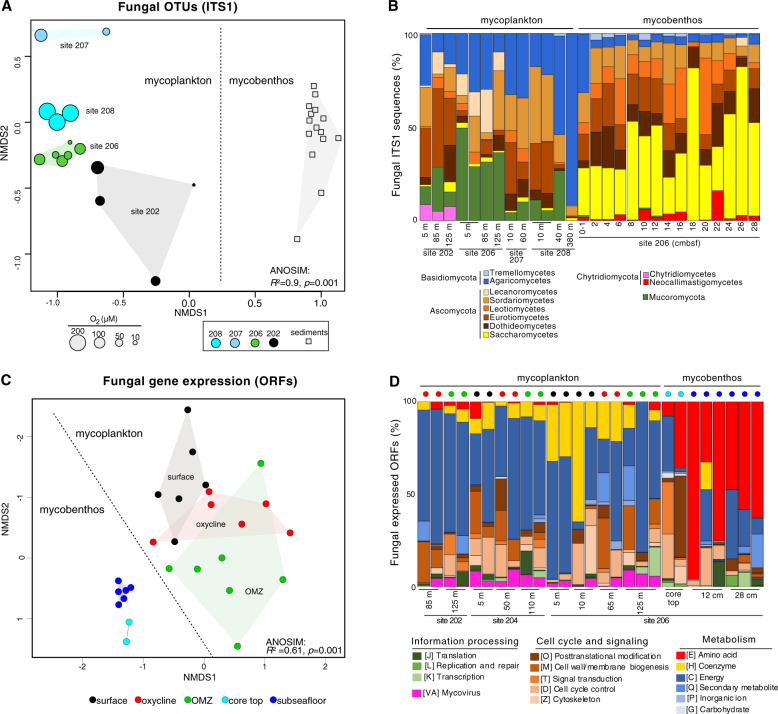
Fungal diversity and gene expression in the water column and sediments. **A** Non-metric multidimensional scaling (NMDS) of samples based on their fungal ITS1 OTU composition. **B** The taxonomic affiliation of fungal ITS1 sequences (from panel **A**). **C** NMDS of samples based on the composition of fungal ORFs in the metatranscriptomes. **D** The functional annotation of fungal ORFs (from panel **C**).

We next profiled the fungal taxa present by ITS1 DNA sequencing (Supplementary Table S2), which showed that each site sampled exhibited its own distinct fungal community (Fig. 4A). Moreover, there was a clear separation of fungal ITS1 DNA sequence datasets between the seawater and seafloor habitats whereby the water column had a higher proportion of fungi ITS1 DNA sequences affiliated with the Agaricomycetes (Basidiomycota) whereas at and below the seafloor the fungal ITS1 DNA sequences are increasingly dominated by those affiliated with the ascomycete yeast group Saccharomycetes (Fig. 4B). Within the anoxic sediments, ITS1 DNA sequences affiliated with the anaerobic fungal group Neocallimastigomycetes [70] were detected throughout the core whereas no ITS1 sequences from this group were detected in the water column (Fig. 4B). Because of systematic biases in the fungal ITS1 annotations due to the underrepresentation of (mostly uncultivated) marine fungi in databases and the ITS1 PCR primers [40, 42], we searched the metatranscriptomes for fungal 18S rRNA to perform phylogenetic analyses and more accurately identify the most active fungal groups.

### Fungal 18S rRNA and gene expression

**Fig. 5:**
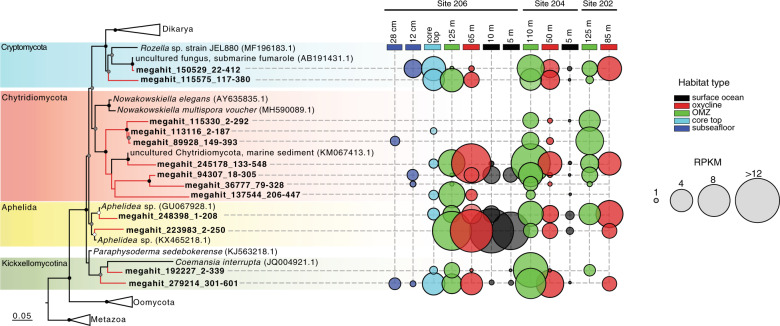
Phylogenetic analyses (PhyML) of 18S rRNA transcripts from metatranscriptomes that are affiliated with fungi together with their closest related sequences from the NCBI-nr database. Circles at nodes show bootstrap support (100 bootstraps, white: >50% support, gray: >75% support, black: >90% support). Fungal 18S rRNA transcripts from the metatranscriptomes are displayed in bold black font, with red coloring on the branch tips. The bubble plot on the right-hand side shows the relative abundance of each fungal 18S rRNA transcript across the different habitats sampled (RPKM: reads per kilobase mapped).

**Fig. 6:**
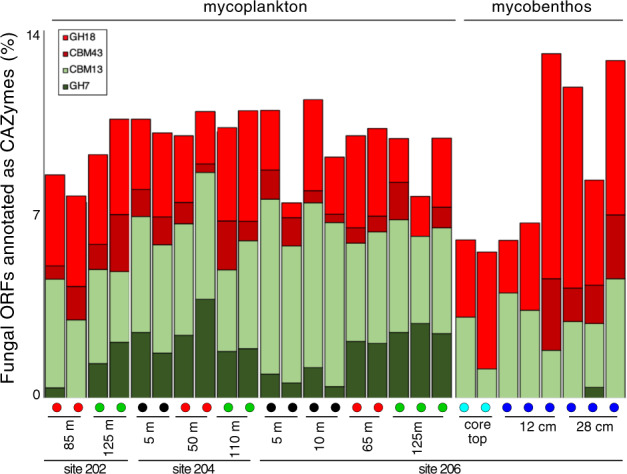
Expression of fungal genes encoding CAZymes in the seawater and sediments. Expression of fungal CAZymes that were significantly different (two-sided *T*-test: *p* < 0.05) between the mycoplankton (GH7, CBM13) and the mycobenthos (GH18, CBM48).

On average, each metatranscriptome sample spanning water column and seafloor habitats (*n* = 27) was sequenced with a depth of 5.9 million reads (SD: 1.5) that amounted to an average of 17,943 contigs per sample (SD: 7,216) (Supplementary Table S2). Ordination of samples based on expressed fungal ORFs showed a grouping of fungal gene expression according to the redox state of the environment, with significantly different fungal expression patterns being found between surface, oxycline, OMZ, core top, and subseafloor habitats (Fig. 4A). The large difference in fungal gene expression between the seafloor and seawater habitats (Fig. 4A) is mostly attributed to a proportionally higher fungal expression of genes involved in energy metabolism in the seawater, compared to the subseafloor where expression of fungal genes is involved in amino acid metabolism dominate in proportional abundance (Fig. 4B). In order to identify the most active fungal groups in the metatranscriptomes, we analyzed the taxonomic composition of 18S rRNA transcripts and their distribution across the water column and sediments. Phylogenetic analysis revealed 18S rRNA transcripts distantly affiliated with the basal fungal groups Cryptomycota [71], Chytridiomycota, *Coemansia* (Kickxellomycotina) [72], and Aphelida (Fig. 5). Mapping of the metatranscriptome reads to the fungal 18S rRNA transcripts revealed that the majority of these basal fungal taxa had the highest relative expression in the OMZ, oxycline, and anoxic seafloor habitats (Fig. 5). The relatively high number of uncultivated Chytridiomycota 18S rRNA transcripts detected (compared to 18S rRNA detected from other fungal groups) was consistent with diverse cytochrome c oxidase transcripts with similarity to the aquatic chytrid genus *Harpochytrium* that were detected primarily in the OMZ metatranscriptomes (Supplementary Fig. S2). The different expression of fungal 18S rRNA between water column and sediments coincided with differential expression of fungal carbohydrate-active enzymes (CAZymes), particularly a proportionally higher expression of fungal glycoside hydrolase family 7 (GH7) encoding transcripts in the water column (Fig. 6).

### ^13^C DNA-SIP of mycoplankton

In order to identify dEPS assimilating fungi in the seawater (mycoplankton), ^13^C-dEPS POM from *C. socialis* was added at a final concentration of 0.2 mg L^−1^, which is ca. three times lower than the average concentration of algal polysaccharides in the Atlantic Ocean [22] and ca. 30% of the DOC concentrations measured in the BUS (0.7 ± 0.1 mg L^−1^; Supplementary Table S3). Therefore, we added the substrate at a concentration within the normal range of dEPS that should be available to the mycoplankton living in the BUS. Within the surface ocean SIP incubations, the oxygen concentrations stayed relatively high between 180–170 µM over the course of the incubations, whereas in the OMZ SIP incubations the oxygen concentrations remained lower throughout the incubation between 80–60 µM (Supplementary Fig. S3) reflecting in situ states of higher and lower dissolved O_2_ from the sampled environments (Fig. 1C).

**Fig. 7:**
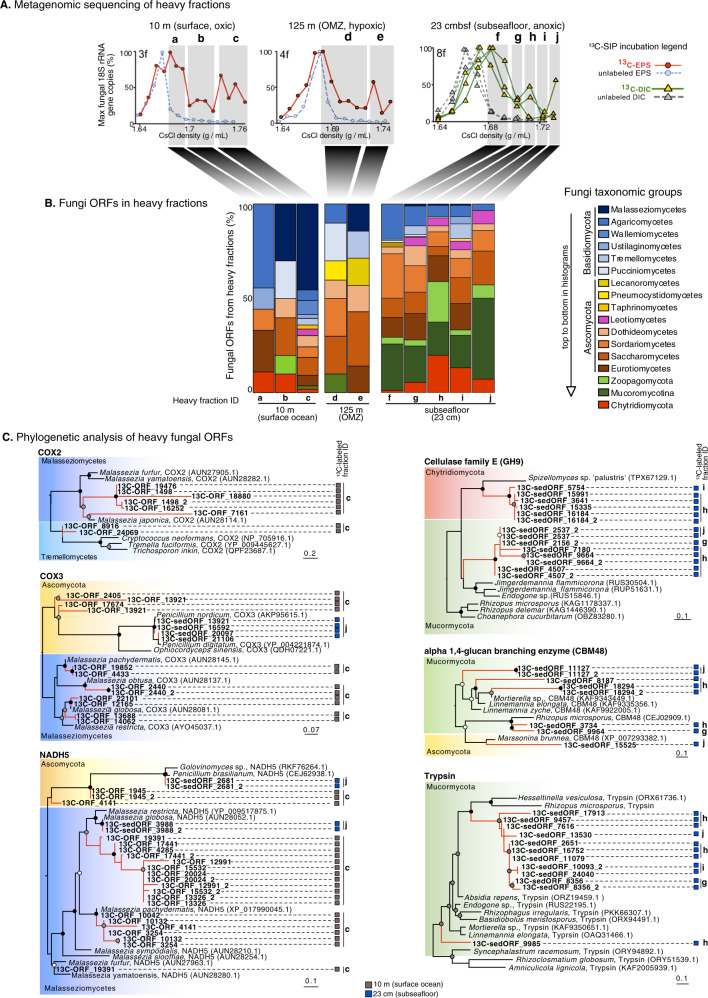
Metagenomic analysis of fungal DNA enriched in heavy SIP fractions from seawater and sediment incubations. **A** Heavy regions of the DNA-SIP density gradients from site 206, which show ^13^C labeling of fungal 18S rRNA genes selected for metagenomic sequencing. **B** Taxonomic affiliation of ORFs in metagenomes from the selected fractions. **C** Phylogenetic analysis of selected fungal ORFs in the ^13^C-labeled metagenomes (bold font) compared to their closest relatives in the GenBank nr database. The ^13^C-enriched fraction in which the ORFs were detected are displayed to the right of the trees. Circles at nodes show bootstrap support (100 bootstraps (white: >50% support, gray: >70% support, black: >90% support). COX2: cytochrome c oxidase subunit 2, COX3: cytochrome c oxidase subunit 3, NADH5: NADH dehydrogenase subunit 5, GH9: CAZY glycoside hydrolase family 9, CBM48: CAZY carbohydrate-binding module 48.

Density gradient fractionated profiles of fungal 18S rRNA gene concentrations show that mycoplankton assimilated more dEPS in the surface ocean compared to the OMZ (Fig. 1E, F) reflecting higher and lower in situ oxygen concentrations, respectively (Fig. 1C). Shotgun metagenomic sequencing of heavy DNA fractions were selected based on a higher relative abundance of fungal 18S rRNA genes in the ^13^C-dEPS incubations at increased buoyant densities compared to the unlabeled controls (Fig. 7A). At 10 m, this included three gradient fractions spanning 1.73–1.76 g mL^−1^ that exhibited a second heavy peak of fungal 18S rRNA genes (Fig. 7A). This second heavy peak of fungal 18S rRNA genes (Fig. 7A) corresponded to an >100x increased relative abundance of fungal sequences in the heavy metagenomes compared to the lighter CsCl densities (Supplementary Fig. S4), a higher diversity of heavy fungal ORFs compared to metagenomes from the lighter CsCl densities (Fig. 7B), and a relatively higher proportion of heavy ORFs with similarity to Malasseziomycetes (Fig. 7B).

Sequencing metagenomes from unlabeled DNA for comparison from the same heavy fractions was not possible because there was so little (or no) rRNA gene copies in the heavier fractions at higher densities where the ^13^C DNA was found to be enriched (Fig. 7A), showing that whatever was sequenced in metagenomes from ^13^C incubation heavy fractions must have become ^13^C labeled. The GC content of the *Malassezia* genome is 31.5% [73], and therefore *Malassezia* DNA should have a density of 1.69 g mL in a CsCl density gradient [74]. Indeed, this falls within the peak of fungal 18S rRNA genes in the unlabeled control that was localized to buoyant densities of 1.66–1.69 g mL. Therefore, finding *Malassezia* ORFs enriched in the heaviest factions with the second peak between 1.73–1.76 g mL in the CsCl gradient (Fig. 7B) shows that these *Malassezia* sequences have been ^13^C labeled and as a result increased their buoyant density from 1.69 g mL to >1.73 g mL.

Heavy metagenomes did not contain any fungal 18S rRNA genes, most likely because the sequencing coverage in the heavy metagenomes was too low (whereas rRNA is in high abundance in transcriptomes and detectable even at relatively low coverage). Therefore, we decided to perform phylogenetic analyses of fungal functional genes from heavy metagenomes to confirm that uptake of ^13^C from fungi had occurred. Phylogenetic analysis of fungal ORFs at 10 m from density fractions exhibiting the second heavy peak of fungal 18S rRNA genes (Fig. 7A) revealed NADH dehydrogenase subunit 5 (NADH5), cytochrome c oxidase subunits II and III (COX2, COX3) deriving from fungal lineages that assimilated the ^13^C-dEPS affiliated with the genera *Malassezia*, *Penicillium*, and *Cryptococcus* (Fig. 7C).

### ^13^C DNA-SIP of seafloor and subseafloor Fungi

Fungal ^13^C assimilation of dEPS was generally lower in the seafloor samples compared to the water column (Fig. 1F). However, the fungi living in the sulfidic subseafloor sediments had ^13^C-EAF values in the ^13^C-bicarbonate SIP incubations that was ca. three times higher compared to carbon assimilation from dEPS (Fig. 1F). The SIP incubations with ^13^C-labeled bicarbonate under anoxic conditions show a clear ^13^C-labeling of fungi 18S rRNA genes compared to unlabeled controls (Fig. 1E) reaching up to 28 atm% (Fig. 1F). O_2_ was below detection for the duration of the entire subseafloor SIP incubations as measured with non-invasive fiberoptic oxygen sensor spots confirming that the subseafloor fungi assimilated carbon under anoxic conditions via anaerobic metabolism. Similar to the seawater SIP incubations, given the potential for co-amplification of fungal non-target eukaryotes with the FF390/FR1 18S rRNA gene primers [46], we performed metagenomics on heavy fractions of the CsCl density gradients that were indicated by qPCR of fungal 18S rRNA genes (Fig. 7A). Heavy SIP metagenomes did not contain any fungal 18S rRNA gene sequences, presumably due to insufficient sequencing coverage. Therefore, we performed a phylogenetic analysis of fungal functional genes (ORFs) that were detected in the heavy metagenomes to confirm that assimilation of ^13^C into fungal DNA had occurred (Fig. 7C). We selected the ^13^C-bicarbonate incubations for metagenome sequencing from the sediment SIP incubations (as opposed to ^13^C-dEPS seafloor incubations) because they showed the highest ^13^C labeling of fungal 18S rRNA genes of all the SIP incubations from sediments (Fig. 1E, F).

In the anoxic ^13^C-bicarbonate incubations with subseafloor sediment, metagenomes from heavy DNA showing ^13^C-assimilation into fungal 18S rRNA genes (Fig. 7A) contain an increased relative abundance of ORFs with similarity to Mucoromycota [75] and Chytridiomycota compared to the water column SIP incubations with ^13^C-dEPS (Fig. 7B). In the sediment SIP incubations, fungal ORFs from the heavy ^13^C-enriched metagenomes with similarity to the polysaccharide degrading CAZyme family GH9 (cellulase family E), the proteolytic digestive enzyme trypsin (serine protease), and the alpha 1, 4 glucans branching enzyme (CAZyme CBM48) were identified (Fig. 7C). Phylogenetic analysis revealed they are affiliated with taxa from Mucoromycota and Chytridiomycota with the closest similarity with the chytrid *Spizellomyces* (Chytridiomycota), *Rhizopus* (Mucoromycota), and *Moriterella* (Mucoromycota) (Fig. 7C).

## Discussion

### Fungal abundance and diversity correlates with DOM profiles

The ca. two orders of magnitude increase in fungal 18S rRNA gene and prokaryotic 16S rRNA gene concentrations at and below the seafloor compared to the water column (Fig. 3C) could help to explain the different DOM compositions between the seawater and sediments. Some sediment DOM could be a product of fungal degradation of more complex particulate substrates in the sediments since marine fungi are often enriched on the particulate organic matter [21]. These differences were also reflected in fungal gene expression (Fig. 4C) and ITS1 DNA sequence beta diversity that were both significantly different between seawater and seafloor (Fig. 4A, B). These results show that communities of marine fungi respond to changing pools of organic matter above and below the seafloor, which translates into a selection for unique communities of fungi in the mycoplankton versus mycobenthos. Similar to subseafloor bacteria [76], our data show that some marine fungi experience selection as they are buried below the seafloor whereas other fungal groups are not present in the water column and therefore might be endemic to the sediment. Selection for fungi in the sediments may be due to the physical properties such as a high density of particles enabling the growth of an increased number of marine fungi that have a particle attached lifestyle [21]. A more densely concentrated seafloor community of fungi (Fig. 3C) that is expressing different CAZymes (Fig. 6) likely helps to create the significantly different DOM compositions between the sediment and water column (Fig. 3A, B) by degrading detrital particulate organic matter that accumulates at the seafloor during sedimentation.

The site-specific mycoplankton communities indicated in the ITS1 DNA data (Fig. 4A) coincided with site-specific diatom composition (Supplementary Fig. S5). Moreover, higher mycoplankton expression of genes involved in coenzyme metabolism in the surface ocean at site 206 (Fig. 4D) coincided with a proportionally higher gene expression in the metatranscriptomes from diatom genera *Pseudo-nitzschia* and *Rhizosolenia* at site 206 (Supplementary Fig. S5). These site-specific communities of diatoms and mycoplankton could be related to host-parasite specificity of fungal-diatom parasitic interactions [19]. Evidence for this is given by the relatively high expression in the surface ocean samples of 18S rRNA from *Aphelidea* at site 206 (Fig. 5), a deeply branching group of intracellular fungal parasitoids of eukaryotic phytoplankton including diatoms [77].

The utilized 18S primers for fungi were shown to co-amplify other eukaryotic sequences, especially in surface Ocean samples [46]. Although this bias might have inflated fungal 18S copy numbers in the surface samples, we still found fungal 18S copy numbers to increase with greater depth and to be the highest in the sediments. Therefore, even if non-target amplification occurred in surface ocean samples, it had no influence on the overall observed trend.

### Basal fungal taxa

No 18S rRNA transcripts were detected in metatranscriptomes with affiliation to Dikarya (Ascomycota or Basidiomycota) (Fig. 5), which is notable considering that Ascomycota and Basidiomycota were abundant in the ITS1 DNA datasets (Fig. 4B). Because the metatranscriptomes detect the active portion of the community, whereas the ITS1 (DNA) detects both active and inactive (dormant) cells, basal fungi are detected in the metatranscriptomes (Fig. 5) appear to be more transcriptionally active compared to Dikarya in the BUS. With the exception of one 18S rRNA transcript affiliated with *Aphelidea* that had high expression in surface ocean waters, most of the basal fungal lineages generally had the highest 18S rRNA expression in the oxycline, OMZ, and anoxic sediment (Fig. 5) indicating a lifestyle suited to low oxygen conditions. In particular, the two fungal 18S rRNA transcripts affiliated with the Cryptomycota [71] were detected exclusively in the oxycline, OMZ, and seafloor samples at Site 206 and detected only with very low coverage in the surface ocean at Site 204 (Fig. 5). This is consistent with the widespread distribution of the highly diverse Cryptomycota group in anoxic sediment habitats [78].

The metatranscriptome data showing that basal fungal lineages have higher activity in anoxic sediments is also supported in the SIP metagenome data. Carbon assimilating fungi in the anoxic sediments were mainly affiliated with Chytridiomycota and Mucoromycota lineages (Fig. 7), both groups that branch deeply within the fungal tree of life, marking the transition toward the more derived Dikarya [72]. The higher relative abundance of fungal sequences affiliated with the seafloor Mucoromycota from the heavy metagenomes was consistent with an increased relative abundance of Mucoromycota transcripts in the subseafloor metatranscriptomes (Supplementary Fig. S6). Mucoromycota, therefore, had relatively high activity in the anoxic subseafloor, and we interpret their lower relative abundance in the subseafloor ITS DNA dataset (Fig. 4B) being due to known ITS1 PCR primers biases against the Mucoromycota [40].

The finding that the carbon assimilating fungi detected in the anoxic sediments were primarily affiliated with Chytridiomycota and Mucoromycota (Fig. 7C), and that all of the detectable 18S rRNA transcripts derive from basal fungal groups (Fig. 5) is consistent with genome evolution studies showing that early fungal evolution involved predominantly aquatic lineages [79]. The relatively high activity of the basal fungi seen here in the OMZ and anoxic sediments would fit with the prediction that fungi evolved in an aquatic Proterozoic environment [80] because Proterozoic atmospheric oxygen concentrations are predicted to have been relatively low compared to modern values reaching concentrations possibly as low as <0.1% of present atmospheric levels [81].

### dEPS assimilating mycoplankton

Because low oxygen conditions were maintained for the OMZ samples during the SIP incubations (Supplementary Fig. S3), the reduced energy availability associated with this condition [67] helps to explain why assimilation of dEPS by the OMZ fungi was lower compared to surface ocean fungi (Fig. 1E, F). However, the higher assimilation of dEPS by fungi in the sunlit surface ocean also coincides with a higher gene expression of diatoms compared to the OMZ (Fig. 1D). The proportion of diatom transcripts steadily decreased in relative abundance from the surface ocean into the subseafloor (Fig. 1D), which is likely due to there being fewer numbers of active diatoms with increasing water depth. Therefore, the extent to which fungi assimilate dEPS across redox stratified seascapes is likely controlled by both oxygen concentrations, as well as the abundance and activity of the diatom community.

Our finding of relatively high ^13^C-EAF values for marine fungi that were assimilating dEPS particles in the water column of the BUS compared to bacteria and archaea (Fig. 1F) may explain why fungi have been previously found to be abundant and active on marine snow particles [21], where EPS from transparent exopolymers derived from diatoms is often abundant [82]. Our results show that fungi are enriched on POM because they have an affinity for the diatomaceous EPS. We used ^13^C-dEPS POM from the diatom *Chaetoceros socialis* which is rich in polysaccharides and “colloidal EPS” [83, 84]. Phylogenetic analysis of Rubisco transcripts confirms that *Chaetoceros* related organisms were active in the BUS at the sites and depths where the DNA-SIP incubations were performed (Fig. 4A), justifying the use of EPS from this organism for the SIP experiments.

*Malassezia* affiliated fungi were well represented in our DNA-SIP dataset as dEPS assimilators from the surface ocean (Fig. 7C), which are a group of facultative yeasts that are widespread in marine habitats [12, 85, 86]. Because it is a major component of the human skin mycobiome the possibility of contamination by laboratory staff should be carefully evaluated [86]. However, as seen with other uncultivated marine *Malassezia* sequences [86], the ^13^C-labeled NADH5, COXII, and COXIII genes affiliated with *Malassezia* are phylogenetically interdigitated amongst *Malassezia* isolated from human hosts and other terrestrial substrates and the heavy *Malassezia* sequences do not coalesce into a monophyletic clade (Fig. 7C). This indicates that the *Malassezia* related fungi that assimilated dEPS in our SIP experiments are not due to human contamination, but are rather derived from the marine environment of the BUS. *Malassezia* is a widespread group of fungi in the ocean [12, 86] and our results indicate they should be considered as important degraders of EPS in planktonic marine environments.

The higher diversity of fungal genes in the heavy fractions of the SIP gradients from 10 m and 125 m seawater incubations (Fig. 7B), and the increased relative abundance of fungal metagenomic reads in heavy SIP fractions (Supplementary Fig. S4), show that fungi are indeed labeled in the SIP incubation and were using the ^13^C-labeled EPS. Therefore, despite the possibility for co-amplification of non-target eukaryotes by the 18S primers [46] fungal DNA was abundant in the heavy SIP fractions which were quantified with our qPCR approach. The identification of *Malassezia* from the SIP metagenomes being involved in the assimilation of carbon from the dEPS is noteworthy considering no fungi from the Malasseziomycetes were detected with the ITS1 DNA dataset (Fig. 4B). This points to the bias in the ITS1 PCR primers that are known to be biased against the Malasseziomycetes [40]. Moreover, transcripts affiliated with *Malassezia* were found only at relatively low (<0.1% of total reads) proportions in the metatranscriptomes, compared to 50% of reads in heavy fraction metagenomes from dEPS SIP incubations at 10 m (Supplementary Fig. S6). *Malassezia*, therefore, had a low in situ activity but became more active in the presence of added dEPS.

### Subseafloor fungi feeding on in situ produced organic matter

The vertical profile of subseafloor fungal 18S rRNA genes indicate stimulation of fungal growth in the nitrate-sulfide transition zone between 10 and 15 cmbsf. This zone of increased fungal growth coincides with an increase in expressed ORFs from known sulfide-oxidizing chemolithoautotrophic bacteria (Fig. 2D). It also coincides with an enrichment in fungal OTUs affiliated with the Erysiphales (Fig. 2C), the majority (> 90%) of which had the closest similarity to the plant pathogenic fungus *Microidium phyllanthi* [87]. Since this area of increased fungal abundance coincides with higher abundances of 16S rRNA gene copies (from bacteria and archaea) and gene expression from chemolithoautotrophic, sulfide-oxidizing bacteria, the subseafloor fungi may be growing in this redox interface by feeding on the necromass of sulfide-oxidizing bacteria (Fig. 2D) that are performing carbon fixation in the absence of light (chemosynthesis).

We tested this hypothesis by analyzing the dark DNA-SIP incubations with ^13^C-labeled bicarbonate, whereby after a 10-day incubation in the dark at 10 °C under anoxic conditions, there was a clear ^13^C-labeling of fungi 18S rRNA genes reaching up to 28 atm% (Fig. 1F). O_2_ was below detection for the duration of the entire incubations as measured with non-invasive fiberoptic oxygen sensor spots confirming that the fungi had assimilated the labeled carbon under anoxic conditions via anaerobic metabolism. Because fungi are heterotrophs, they could only have assimilated the ^13^C-label in these dark incubations via feeding on autotrophic organisms that fixed the inorganic labeled carbon via chemosynthesis (and thereby assimilated the labeled bicarbonate into ^13^C-organic matter). Fungal decomposers of bacteria have been identified in other DNA-SIP studies, for example from fungal genes in ^13^C-enriched nucleic acid fractions revealing that fungi decompose methylotrophic bacteria in rice field soil [88]. Similarly, the enrichment of heterotrophic fungal 18S rRNA genes in the heavy CsCl fractions from the ^13^C-labeled bicarbonate incubations indicate anaerobic fungal decomposition of chemosynthetic bacteria that were fixing the labeled bicarbonate.

Next, we determined the presence of active sulfide-oxidizing chemosynthetic taxa affiliated with the Gammaproteobacteria via phylogenetic analysis of the DsrA/B transcripts from the metatranscriptomes (Supplementary Fig. S7). Following this, we then confirmed that sulfide-oxidizing chemolithoautotrophic bacteria were indeed fixing the labeled bicarbonate. This was done by searching the previously published [28] 16S rRNA gene qSIP dataset for ^13^C-bicarbonate assimilating OTUs affiliated with known chemolithoautotrophic groups. Bacterial OTUs affiliated with the Desulfobulbaceae, a group of chemolithoautotrophic cable bacteria [89], had the highest levels of ^13^C-enrichment in the anoxic ^13^C-bicarbonate incubations (Supplementary Fig. S8). Moreover, ^13^C-assimilation by OTUs affiliated with the chemolithoautotrophic sulfur-oxidizing Gammaproteobacteria Ectothiorhodospiraceae and Thiotrichaceae [90] were also detected (Supplementary Fig. S8). The relative expression of ORFs from these groups peaked within the sulfide-nitrate transition zone where fungal 18S rRNA gene concentrations increased (Fig. 2D). These results demonstrate that autotrophic chemosynthetic bacteria form the base of an anaerobic feeding chain in the BUS, whereby saprotrophic fungi feed on organic carbon produced in situ via chemosynthesis. We assume that the fungi forage and decompose mainly dead cells (‘‘necromass’’) that became ^13^C-labeled while they were alive, but it may be possible that some of the fungi decompose living chemolithotrophic bacteria, or some fungi could thrive on released compounds (DOM) and exometabolites released from living bacteria. Our data are not able to differentiate between possibilities, but do show subseafloor Fungi feeding on in situ produced organic matter derived from chemosynthesis. The amount of chemosynthesis is apparently sufficient to fuel the net growth of the foraging fungi within the nitrate-sulfide zone (Fig. 2B). However, we acknowledge that other autotrophic organisms (other than S-oxidizers) could also be contributing to the primary production below the seafloor and we cannot attribute all of the CO_2_ fixations to only S-oxidizers.

Unlike the fungal ORFs from ^13^C-enriched metagenomes from the surface ocean that were dominated by *Malassezia*, *Malassezia*-affiliated ORFs are notably lacking in the subseafloor ^13^C-enriched metagenomes (Fig. 7B, C) and metatranscriptomes (Supplementary Fig. S6) indicating a selection for fungi that preferentially assimilate carbon produced in situ below the seafloor. The subseafloor fungi had higher ^13^C-EAF values in the ^13^C-bicarbonate SIP incubations (from feeding on chemosynthetic bacteria) that was ca. three times higher compared to subseafloor fungal assimilation from dEPS (Fig. 1F). This indicates that many subseafloor fungi, including those associated with the Mucoromycota and Chytridiomycota (Fig. 7C), prefer to assimilate chemosynthetic necromass over dEPS. Thus, it appears that the sulfide-nitrate redox transition zone fuels a zone of chemosynthetic primary production, which in turn supports fungi that forage the resulting organic carbon under anoxic conditions. Dark carbon fixation proceeds at rates up to 370 Tg C/year [91] fueled by sulfide oxidizing bacteria [90], and our results demonstrate a direct link between subseafloor fungi and the turnover of this fixed carbon. Taken together, our findings show that subseafloor fungi of the BUS subsist under anoxic conditions on organic matter produced via chemosynthesis, as opposed to the mycoplankton living in the surface ocean and OMZ whose niche is controlled to a larger extent by the activity of diatoms and availability of dEPS.

### Protein and organic nitrogen degrading fungi in the subseafloor

The fungal protein degrading trypsin genes and CBM48 genes from the heavy fractions of the subseafloor SIP incubation had the closest phylogenetic affiliation to Mucoromycota, particularly those from *Rhizopus* (Fig. 7C). This was consistent with the dominance of fungal transcripts below the seafloor being affiliated with the Mucoromycota (Supplementary Fig S5). Due to their production of proteolytic enzymes, *Rhizopus* fungi are often used in “tempe-type” fermentation of plant (e.g., soybean) protein [92]. Trypsin is an enzyme used by Fungi for lysis of protein substrates [93] and thus the ^13^C-labeling of *Rhizopus* trypsin-encoding genes suggests that protein fermenting Mucoromycota actively decompose chemolithoautotrophic bacteria in the sulfidic Namibian subseafloor. A subseafloor fungal diet based on protein fermentation could be explained by the higher proportion of organic *N* substrates in the sediment DOM (Supplementary Fig. S1).

The highest proportion of N-containing DOM compounds were detected at the core top, suggesting a larger contribution of relatively young DOM in pore waters of the surface sediments. The increased N-containing DOM may also be due to the degradation of organic matter by a wider spectrum of active enzymes that are present in surface sediments [94]. Moreover, the decrease in abundance of unsaturated compounds with N at the subseafloor (>10 cmbsf) (Supplementary Fig. S1) suggested that those DOM compounds were highly reactive and rapidly reworked during a degradation continuum increasing with sediment depth [95]. The increased abundance of amino acid carbon and peptide-like substrates in the sediment pore-water DOM pool (Supplementary Fig. S1) can be partly explained by a significantly higher mycobenthos expression of CAZymes GH18 and CBM43 (two-sided *T*-test: *p* = 0.003, Figs. 6, Supplementary  S9), that are involved in the degradation of N-containing organic matter such as peptidoglycan and amino sugars [54]. The different composition of sediment and seawater DOM (Fig. 3A, B) can therefore be partly attributed to different fungal enzymes expressed differently in both environments. A subseafloor fungal diet rich in organic nitrogen is furthermore reflected in the increased expression of fungal amino acid transport and metabolism genes in the sulfidic subseafloor sediments (Fig. 4D). The unique expression of seafloor fungal CAZymes (Supplementary Fig. S9) likely help to create the unique composition of DOM in sediment pore water, compared to the seawater DOM (Fig. 3A, B).

### Trends in microbial carbon assimilation from surface ocean to subseafloor

A general trend was observed, whereby dEPS assimilation by both prokaryotes (bacteria and archaea) and Fungi were highest in the surface ocean and OMZ samples and decreased by several orders of magnitude in the anoxic sediments below the seafloor (Fig. 1F). This data supports the ecological theory that decreased availability of O_2_ for aerobic metabolism results in lower trophic transfer efficiency of carbon across trophic levels (e.g., from primary producers to secondary producers) in anoxic environments [96]. However, an alternative explanation is that dEPS may be at a lower concentration in the subseafloor compared to the surface ocean (where it is produced) and thus a dEPS assimilating seafloor microbial community is not selected or acclimated to decompose dEPS, and as a result, it is assimilated more slowly.

Calculating ^13^C-EAF values for fungi (Fig. 1F) based on rRNA genes may be biased by the possibility for co-amplification of non-target eukaryotes by the 18S primers which may influence the fungal quantification [46], and therefore the fungal EAF values. Thus, we interpret the ^13^C-EAF values for the marine fungi (Fig. 1F) with caution. However, fungi have increased relative abundance in heavy SIP metagenomes from CsCl densities where qPCR with fungal 18S rRNA primers indicates fungal ^13^C-labeling (Supplementary Fig. S4), showing that fungi indeed assimilated the ^13^C label. Future quantification of marine fungi with the FF390/FR1 primers should include blocking primers of non-target groups as this greatly reduces the co-amplification of non-targets in marine samples [46]. This should allow for more accurate carbon assimilation estimates of marine fungi using qSIP [64], and a comparison against carbon assimilation performed by heterotrophic bacteria and archaea.

### Comparing microbial CAZyme expression

**Fig. 8:**
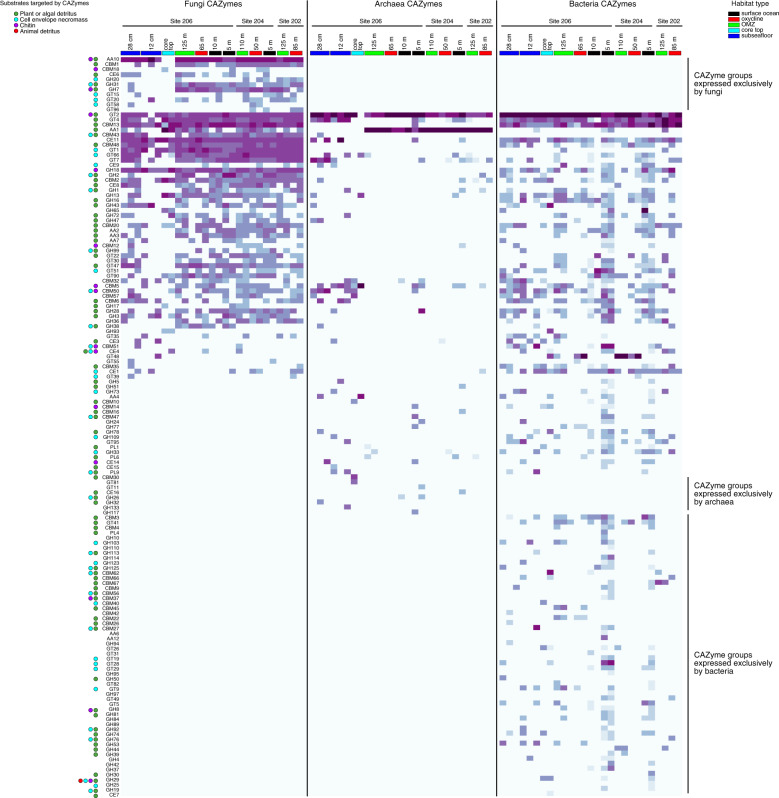
Heatmap showing relative abundance of expressed CAZymes with closest similarity to fungi, bacteria, or archaea. The heatmap colors are scaled to the relative abundance of total CAZyme hits per group per sample, representing the proportional abundance per column in the heatmap (lighter blue = lower, darker purple = higher).

In order to better understand the enzymatic diversity of fungi versus prokaryotes (bacteria and archaea) in the BUS that are potentially involved in organic matter turnover, we compared expressed CAZymes [54] affiliated with fungi, bacteria, and archaea across all metatranscriptomes (Fig. 8). This revealed that fungi expressed 12 groups of CAZymes that were not expressed by bacteria or archaea in any of the samples. Of particular note are the CAZyme groups AA10, CBM1, CBM18, GH7, and CE6 that are expressed exclusively by fungi. The enzymes within these CAZyme groups have been shown to act on chitin, hemicellulose, xylan, endo-*β*-1,4-glucans, and cellulose [54]. This unique profile of fungal CAZyme expression (Fig. 8) indicates that the marine fungi we detected in the BUS are potentially playing a role in the degradation of cellulose, hemicellulose, and/or chitin.

The diversity of expressed fungal glycoside hydrolases (GH) was notably higher in the seawater column compared to the sediments, and expression of several GHs (GH20, GH47, GH38) was detected only in the seawater (Supplementary Fig. S9). These differently expressed fungal GHs in the water column likely play a role in creating the unique DOM composition of the water column compared to the sediments (Fig. 3A, B). Most of the fungal transcripts encoding GH7 CAZymes were detected in the water column (Figs. 6, Supplementary  S9), which coincided with the high proportion of saturated O-rich DOM compounds (Supplementary Fig. S1) and higher fungal assimilation of ^13^C-dEPS compared to the sediments (Fig. 1F). Within the water column, fungal GH7 enzymes had the highest expression in the OMZ and oxycline (Fig. 6) and therefore likely help to degrade sinking algal biomass within low oxygen marine environments and OMZs. Phylogenetic analysis of fungal GH7 transcripts revealed similarities to cellobiohydrolases encoded by fungal taxa affiliated with *Daldinia, Didymella, Schiophyllum*, and *Trametes* (Supplementary Fig. S2). Cellobiohydrolases of the GH7 family remove cellobiose from the ends of the cellulose polymer [54], marking a critical preliminary step in the hydrolysis of cellulose into small oligosaccharides and ultimately glucose [97]. Fungal GH7 enzymes in marine surface waters and OMZs therefore may help to convert cellulose into monosaccharides and oligosaccharides that then become accessible to the larger microbial community. The differential expression of fungal GH7 between the seawater and sediment habitats (Fig. 6) likely plays a role in determining the different compositions of DOM in the pelagic and benthic habitats (Fig. 3A, B). Since no cellobiohydrolase transcripts were detected from bacteria or archaea fungal cellobiohydrolases may be particularly important for the enzymatic degradation of cellulose in the ocean, which is rare in the marine environment compared to terrestrial ecosystems where cellulose is the major polysaccharide [94].

These results could motivate future studies to examine whether the unique fungal CAZyme expression seen here translates into a distinct ecological role for fungi in the degradation of complex organic matter in marine environments compared to bacteria and archaea. The distinct CAZyme expression profiles between bacteria and fungi (Fig. 8), together with a comparable amount of dEPS assimilation by bacteria and fungi in several samples (Fig. 1F) suggest the possibility that bacteria and fungi may work together to degrade marine POM by each producing a unique suite of digestive enzymes. Future studies could investigate whether marine fungi compete with bacteria for the same substrate components or whether the relationship is more cooperative.

## Conclusion

Our findings show that marine fungi play a quantitatively relevant role alongside heterotrophic prokaryotes bacteria and archaea in the cycling of marine organic matter in the water column and sediments. The combined gene expression and SIP results provide insights into the biological mechanisms underlying relatively high rates of marine fungal carbon assimilation. Our findings highlight the importance of heterotrophic marine fungi alongside heterotrophic bacteria and archaea in the marine carbon cycle. The data show that fungi not only degrade diatom derived organic matter sinking from the water column, but that distinct subseafloor fungi are specialized on organic matter that is produced in the sediments. These differences may define the different niches occupied by the mycoplankton and mycobenthos, leading to phylogenetically unique communities of fungi across redox stratified marine ecosystems. Marine fungi are shown here to affect the diversity and composition of marine DOM across seawater and seafloor habitats, and thereby help to structure carbon flow from primary producers in the ocean.

## Supplementary information

Supplementary information

## Data Availability

Sequence data are publicly available through NCBI BioProject PRJNA525353 with the following accession numbers: SAMN17911873- SAMN17911901 (water column fungi ITS1), SAMN17914346- SAMN17914359 (sediment fungi ITS1), SAMN11047492 - SAMN14752199 (sediment metatranscriptomes), SAMN17911811- SAMN17911835 (water column metatranscriptomes), SAMN17914379- SAMN17914388 (heavy metagenomes from SIP).

## Code availability

All code used has posted on GitHub (https://github.com/williamorsi/MetaProt-database). The MetaProt database is publicly available as a single 32GB amino acid fasta file (10.5282/ubm/data.183). Assembled contigs are available online (10.5282/ubm/data.187).
