# Supplementary material for: Carbon assimilating fungi from surface ocean to subseafloor revealed by coupled phylogenetic and stable isotope analysis
Source: ISME J. 2021 Dec 11;16(5):1245–61. doi: 10.1038/s41396-021-01169-5 (PMC9038920; doi:10.1038/s41396-021-01169-5)
Supplement: Supplementary file 1 — Supplementary information [file 41396_2021_1169_MOESM1_ESM.pdf]

Supplemental Information

Carbon assimilating fungi from surface ocean to subseafloor revealed by coupled phylogenetic and stable isotope analysis

William D. Orsi[1,2*], Aurele Vuillemin[1], Ömer K. Coskun[1], Yanik Oertel[3], Jutta Niggemann[3], Volker Mohrholz[4], Gonzalo V. Gomez-Saez[3,5]

[1]Department of Earth and Environmental Sciences, Paleontology & Geobiology, Ludwig-Maximilians-Universität München, Richard-Wagner-Strasse 10, 80333 Munich, Germany.

[2]GeoBio-CenterLMU, Ludwig-Maximilians-Universität München, Richard-Wagner-Strasse 10, 80333 Munich, Germany.

[3]Research Group for Marine Geochemistry (ICBM-MPI Bridging Group), Institute for Chemistry and Biology of the Marine Environment (ICBM), University of Oldenburg, Oldenburg, Germany.

[4]Baltic Sea Research Institute, Warnemünde, Seestrasse 15, D18119 Rostock, Germany

[5]Alfred Wegener Institute, Helmholtz Centre for Polar and Marine Sciences, Bremerhaven, Germany.

Table S1. Sequencing statistics for fungal ITS1.

Station	Sample type	Depth (m)	Replicate	# ITS reads	Total ITS OTUs	# fungal OTUs annotated to class level against UNITE
site-202	water column	5		122	69	35
		85	a	16,336	200	42
			b	10,757	41	17
		125		13,147	162	42
site-206	water column	10	a	5,939	279	72
			b	11,625	204	54
		65		1,457	194	48
		125		2,052	166	40
	sediment	0-1 cmbsf		26,257	215	59
		2 cmbsf		31,734	315	121
		4 cmbsf		26,267	253	109
		6 cmbsf		23,532	237	85
		8 cmbsf		22,623	247	90
		10 cmbsf		22,776	240	75
		12 cmbsf		11,729	172	57
		14 cmbsf		10,337	197	76
		16 cmbsf		4,992	214	65
		18 cmbsf		469	32	21
		20 cmbsf		17,448	188	61
		22 cmbsf		30,363	161	70
		24 cmbsf		13,031	171	80
		26 cmbsf		11,773	174	25
		28 cmbsf		40,976	144	52
site-207	water column	10		2,283	111	45
		60		4,531	126	45
site-208	water column	10	a	8438	163	61
			b	25,177	106	50
		40		12,228	159	48
		145		8,092	43	19
		380		7,220	116	29

Table S2. Sequencing and assembly statistics for the metatranscriptomes.

Station	Sample type	oxygen level	Depth (m)	Replicate	# of reads (millions)	# of contigs	# reads mapping to contigs	% of reads mapping to contigs	# ORFs annotated
site-202	water column	oxycline	85	a	3	13,274	2.1	70.0	1470
				b	3.6	27,862	2.2	61.1	1901
		OMZ	125	a	4.7	19,254	3.5	74.5	1834
				b	3.4	15,817	2.5	73.5	1419
site-204	water column	Surface (oxic)	5	a	10.2	67,010	8.2	80.4	10038
				b	9.1	41,408	7	76.9	5318
		oxycline	50	a	9.4	21,960	8.2	87.2	3205
				b	7.6	17,647	6.6	86.8	2402
		OMZ	110	a	13.8	27,168	12.2	88.4	3932
				b	7.9	29,864	6.2	78.5	6439
site-206	water column	surface (oxic)	5	a	5.7	19,334	4.6	80.7	7851
				b	6.7	37,547	5.1	76.1	7683
			10	a	7.2	20,735	6.3	87.5	2163
				b	3.7	10,464	3.2	86.5	8074
		oxycline	65	a	7.2	16,298	6.1	84.7	2038
				b	3.5	9,860	2.7	77.1	1241
		OMZ	125	a	1.7	5,526	1.3	76.5	718
				b	5.3	16,420	4.2	79.2	2378
				c	7.7	25,788	6.1	79.2	3362
	sediments	hypoxic	core top	a	4.6	2,602	3.7	80.4	897
				b	11.1	2,927	9.2	82.9	926
		sulfidic	12 cm	a	3.8	4,362	2.7	71.1	1462
				b	2.2	2,726	1.3	59.1	752
				c	3.4	5,888	2.1	61.8	1458
			28 cm	a	3.8	7,429	2.3	60.5	2266
				b	5.7	9,636	4.2	73.7	4087
				c	4.1	5,660	2.7	65.9	3148

Table S3. Physico-chemical parameters of the DOM samples analyzed, including depth, oxygen, dissolved organic carbon (DOC), solid-phase extractable DOC (SPE-DOC) and total amount of molecular formulas identified by FT-ICR-MS per sample. Being "-" not analyzed. Additional information is shown in Figure 3 and Fig. S1.

Station	Sample type	Habitat type (Fig. S2)	Depth (m or cmbsf)	Oxygen (µM)	DOC (µM)	SPE-DOC (µM)	# total FT-ICR-MS formulas
site-202	water column	surface	10	153	69	25	2243
site-202	water column	oxycline	50	147	58	26	2552
site-202	water column	oxycline	75	114	58	32	2473
site-202	water column	OMZ	85	47	60	26	2617
site-202	water column	OMZ	90	19	59	24	2436
site-202	water column	OMZ	120	25	58	22	2571
site-204	water column	surface	15	147	55	24	2424
site-204	water column	oxycline	35	107	56	24	2752
site-204	water column	oxycline	50	98	55	26	2511
site-204	water column	oxycline	75	100	58	24	3114
site-204	water column	oxycline	90	99	59	25	3074
site-204	water column	OMZ	105	59	59	26	3003
site-206	water column	surface	20	116	56	27	2878
site-206	water column	oxycline	40	81	57	25	2605
site-206	water column	OMZ	60	45	57	25	2849
site-206	water column	OMZ	75	27	53	27	2938
site-206	water column	OMZ	90	25	56	27	2752
site-206	water column	OMZ	120	26	57	25	2506
site-206	sediments	Core top	3	-	-	116	2185
site-206	sediments	Core top	5	-	-	120	2470
site-206	sediments	Subseafloor	10	-	-	97	2531
site-206	sediments	Subseafloor	15	-	-	106	2007
site-206	sediments	Subseafloor	20	-	-	144	1700
site-206	sediments	Subseafloor	25	-	-	66	2488
site-206	sediments	Subseafloor	30	-	-	130	2243

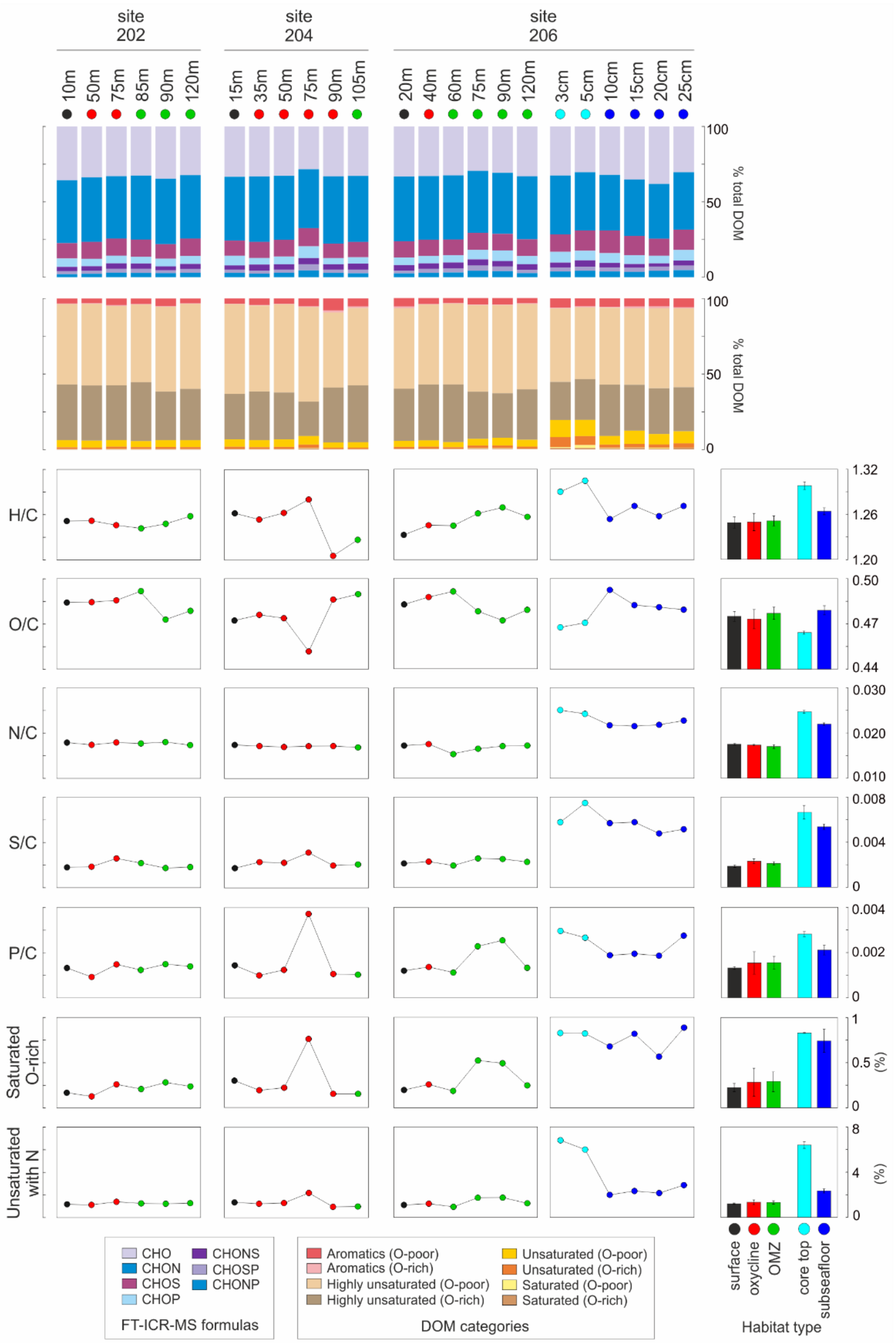

Figure S1: Molecular characterization of dissolved organic matter (DOM) in the BUS water column and sediments. From above to below: relative abundance of the different groups of molecular formulas identified in FT-ICR-MS, relative proportion in total FT-ICR-MS spectra of the different DOM categories, vertical distribution of the DOM differentiating between station and habitat type, with the mean average and standard deviations of the different molar ratios (H/C, O/C, N/C, S/C, P/C) and two selected formulas subgroups (saturated O-rich, former "sugars", and unsaturated compounds with N, former "peptides"). Color code classifies the habitat type: surface (black), oxycline (red), OMZ (green), core top (light blue) and subseafloor (dark blue).

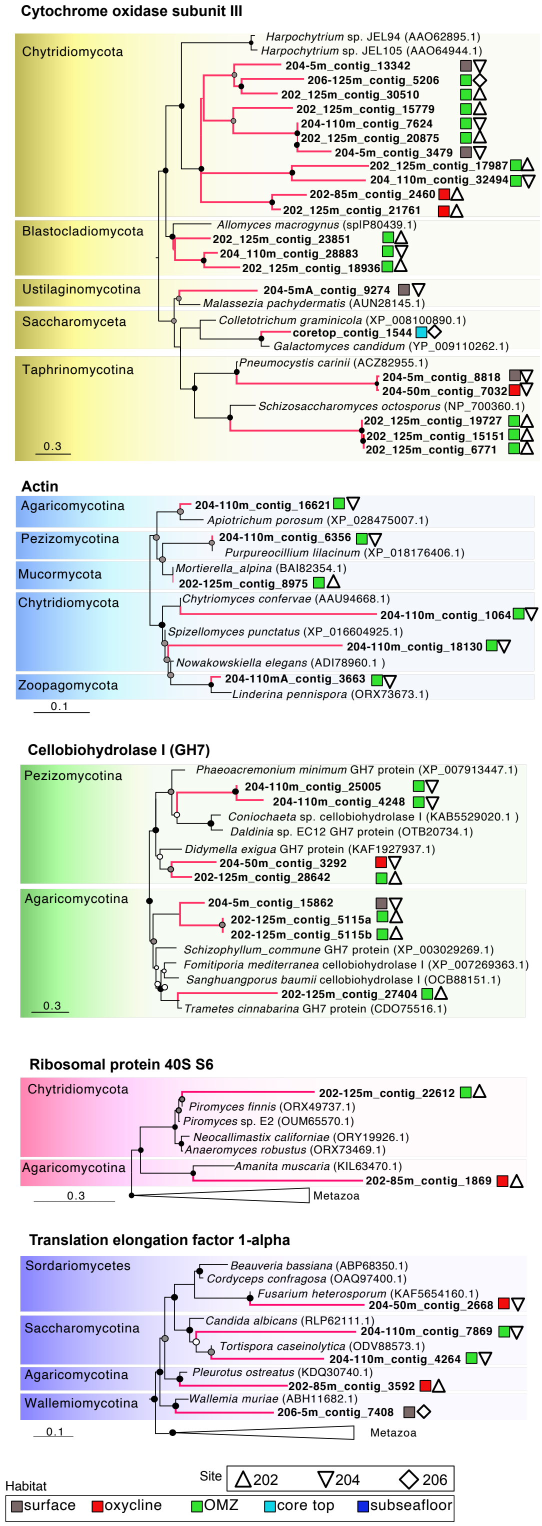

Figure S2. Phylogenetic analyses (PhyML) of expressed ORFs (amino acids) from the metatranscriptomes that are affiliated with Fungi together with their closest related sequences from the NCBI-nr database. Circles at nodes show bootstrap support (100 bootstraps (white: >50% support, grey: >70% support, black: >90% support). Expressed ORFs from the metatranscriptomes are displayed in bold black font, with red coloring on the branch tips. Symbols at the branch tips show at which site and depth the ORF was detected.

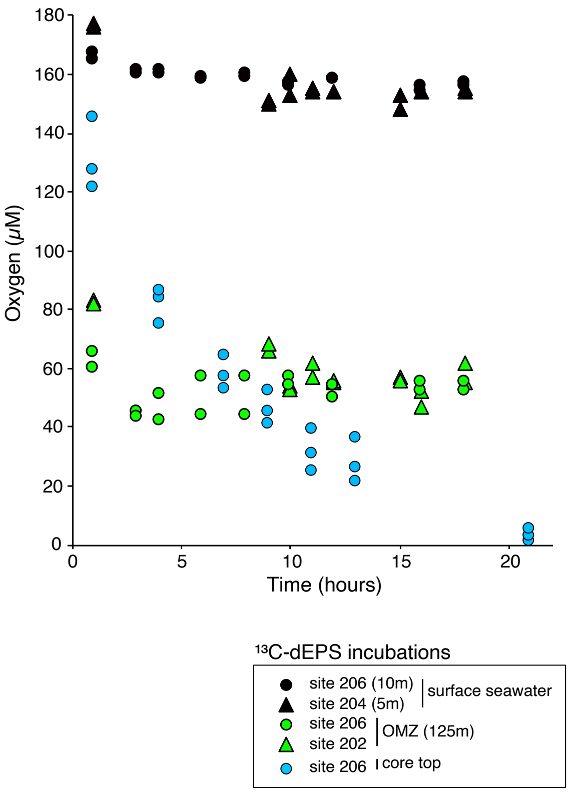

Figure S3 (A) Oxygen consumption during the incubations in the water column and core top. The core top incubation went anoxic after 20 hours and remained anoxic for the remainder of the 10-day incubation.

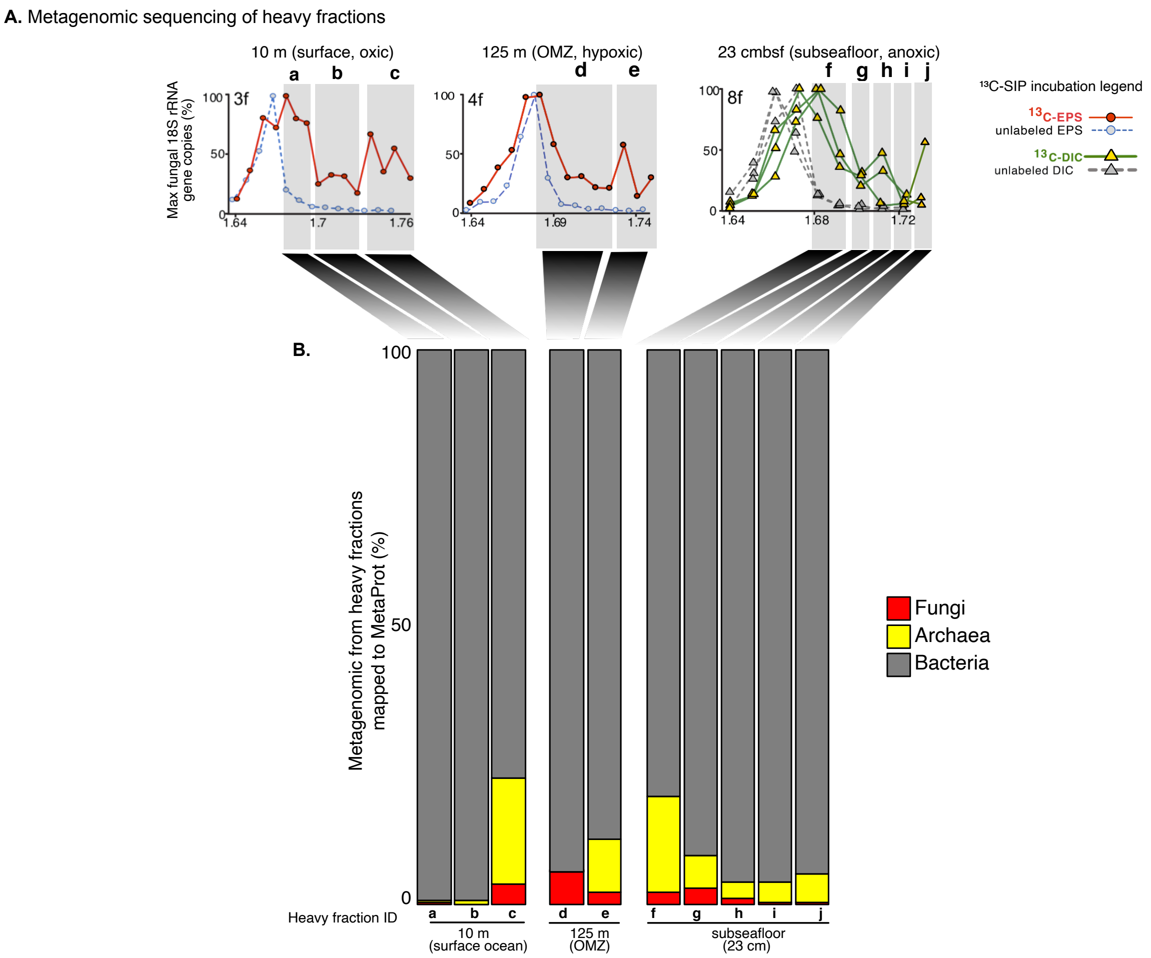

Figure S4. (A) Heavy regions of the DNA-SIP density gradients from site 206, which show ^{13}C labeling of fungal 18S rRNA genes selected for metagenomic sequencing. (B) Proportional abundance of taxonomic affiliations of raw reads in metagenomes from the selected fractions, after BLASTx searches of raw metagenomic reads against the MetaProt database..

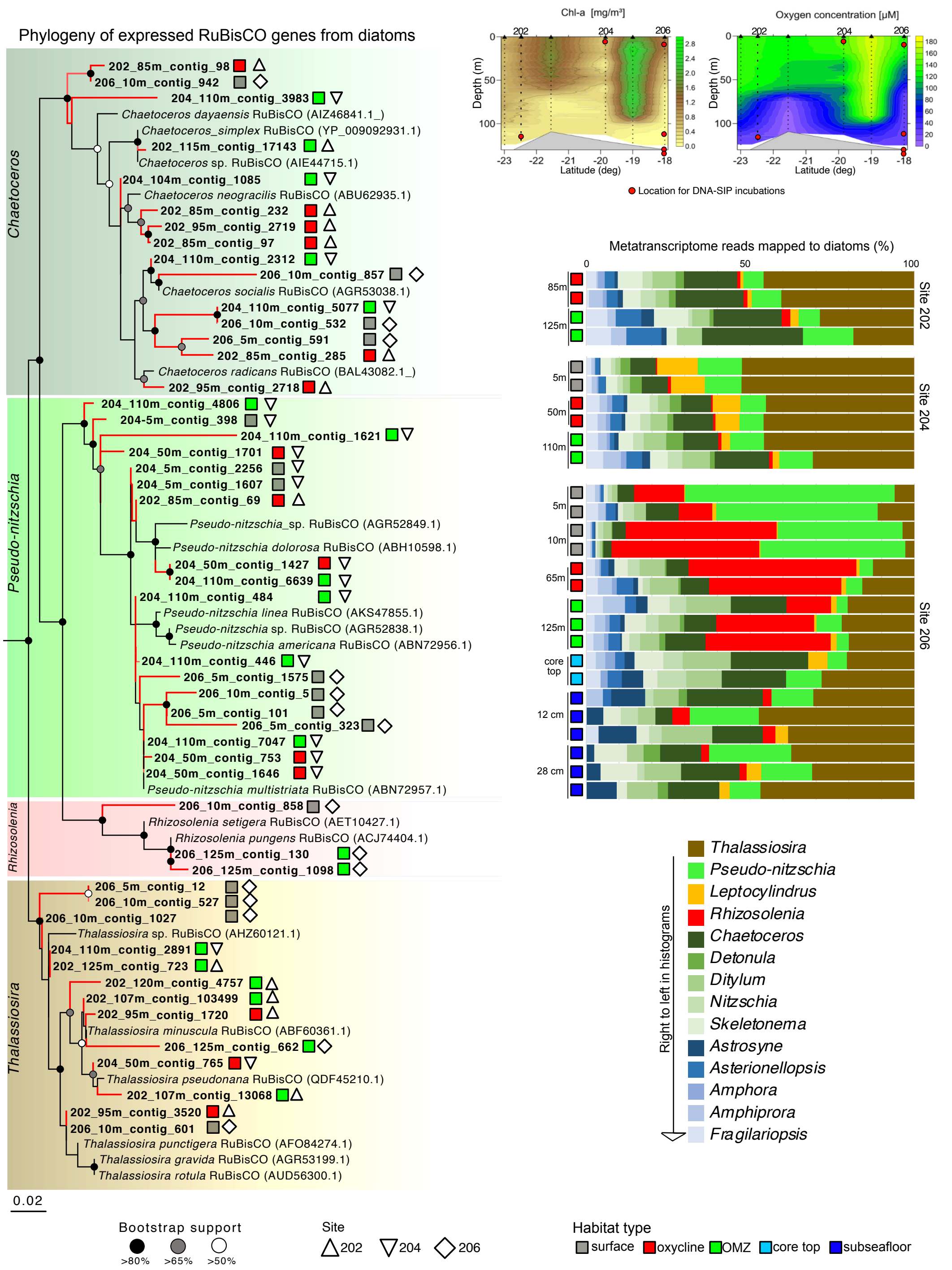

Figure S5: Left: PhyML analysis of expressed RuBisCO ORFs (amino acids) from diatoms in the metatranscriptomes together with their closest related sequences from the MMETSP database (Keeling et al 2014). Expressed ORFs from the metatranscriptomes are displayed in bold black font, with red coloring on the branch tips. Circles at nodes show bootstrap support (100 bootstraps (white: >50% support, grey: >70% support, black: >90% support). Right: Stacked histograms summarizing the relative abundance of ORFs with similarity to diatom groups at the different depths and sites in the BUS. Note the increased expression of ORFs from the genera *Pseudo-nitzschia* (green) and *Rhizosolenia* (red) at site 206.

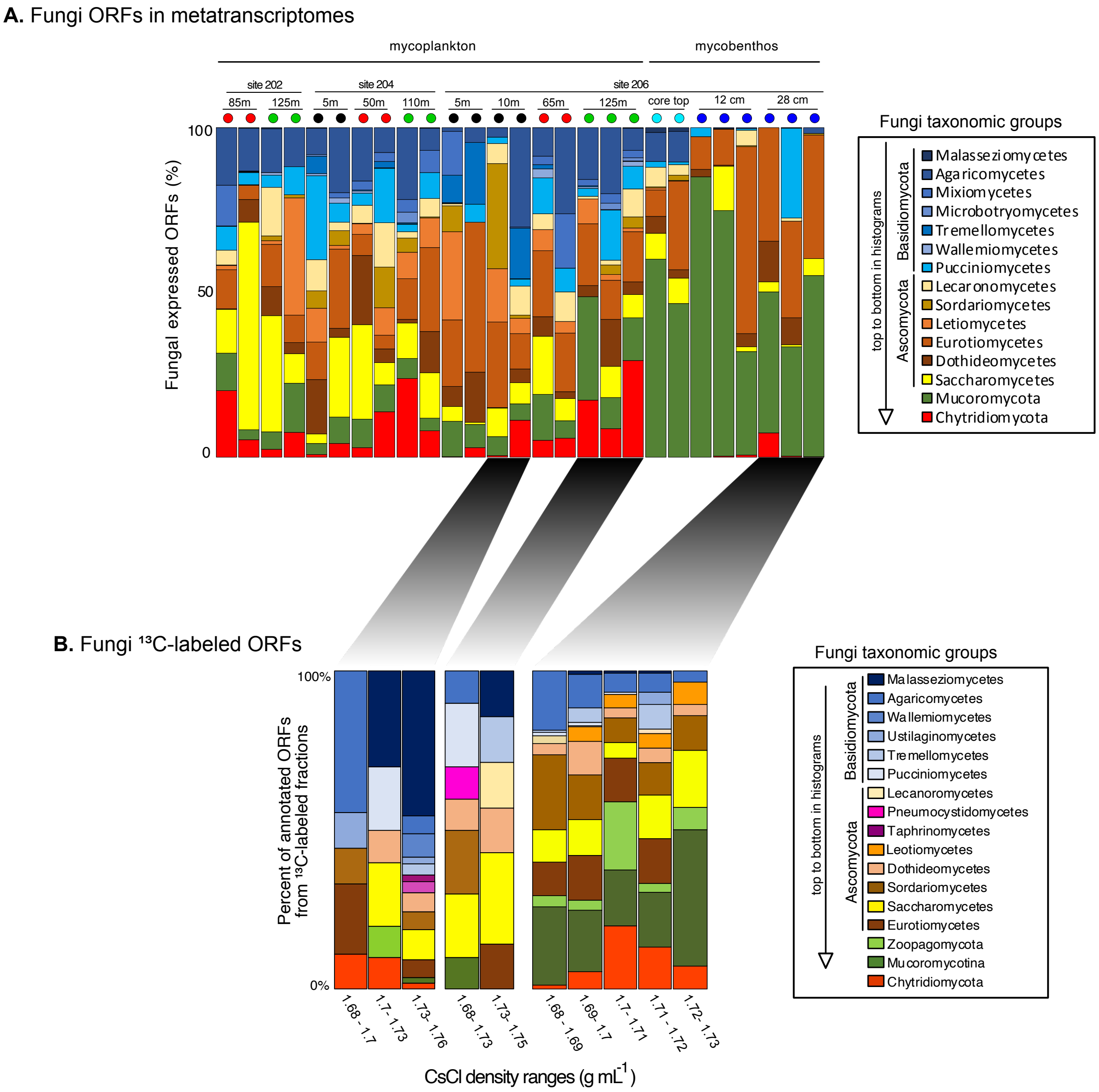

Figure S6. (A) Relative abundance of ORFs with similarity to fungal groups in the metatranscriptomes. (B) Relative abundance of ORFs with similarity to fungal groups in the SIP heavy metagenomes. Grey lines connect the samples from which there exist both metatranscriptome data and SIP metagenome data from the heavy fractions.

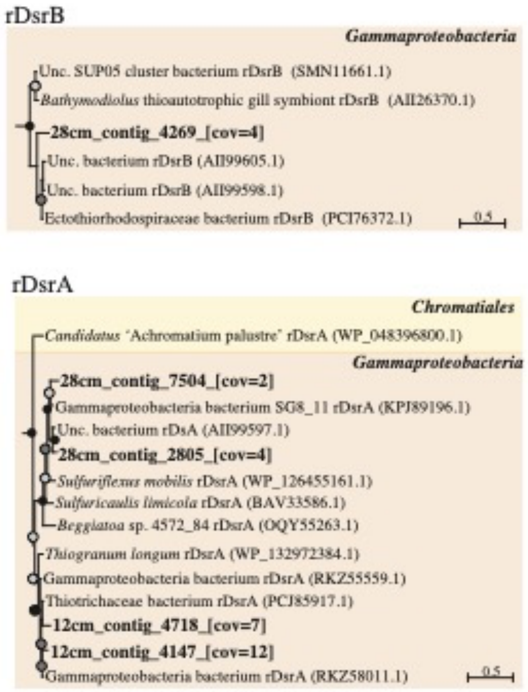

Figure S7. Phylogenetic analyses (PhyML) of expressed ORFs (amino acids) from the metatranscriptomes that are affiliated with rDsrA and rDsrB together with their closest related sequences from the NCBI-nr database. Circles at nodes show bootstrap support (100 bootstraps (white: >50% support, grey: >70% support, black: >90% support). Expressed ORFs from the metatranscriptomes are displayed in bold black font, with red coloring on the branch tips.

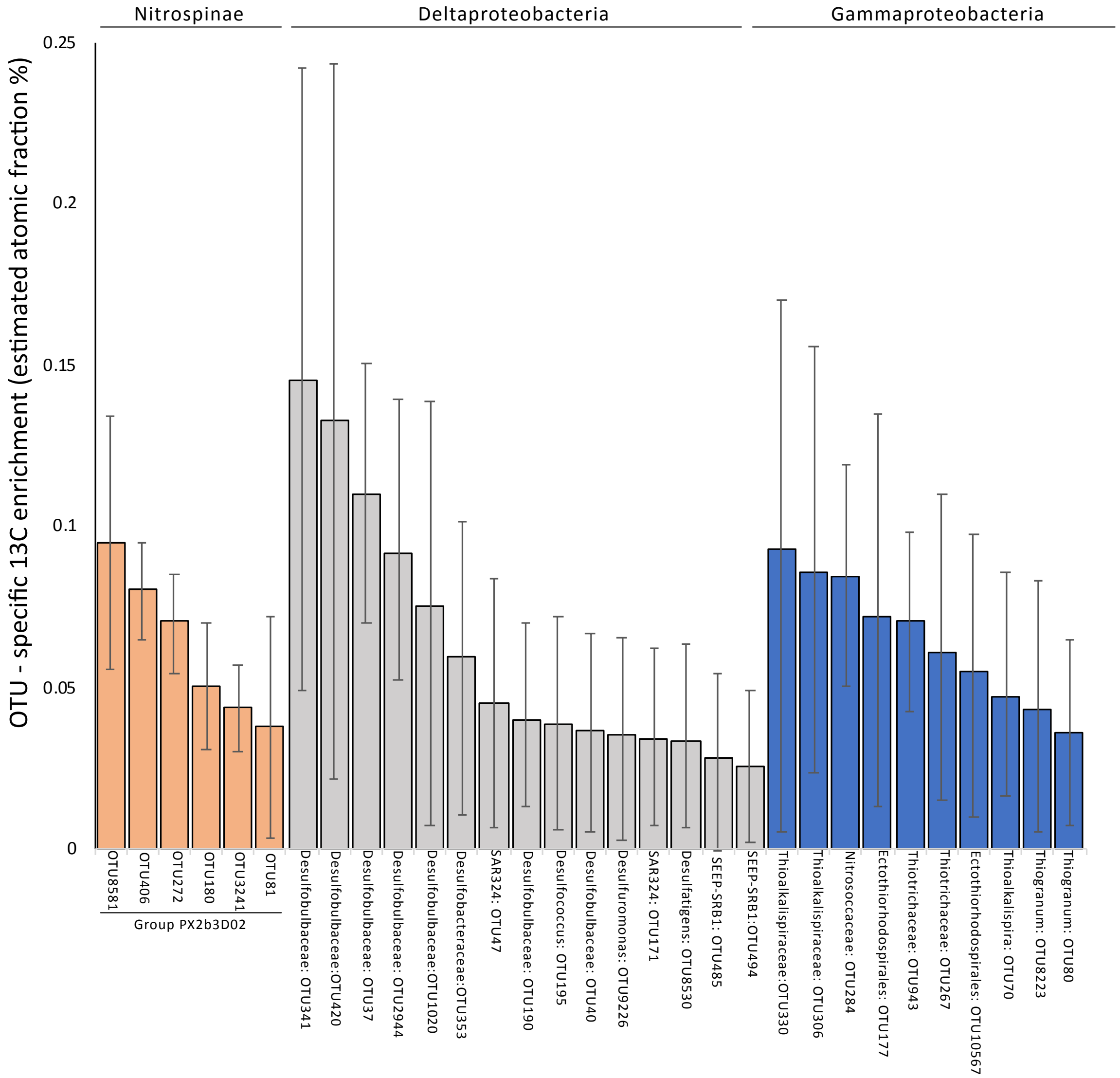

Figure S8. qSIP analysis of 16S rRNA gene OTUs affiliated with known chemolithoautotrophic groups displaying a significant ^{13}C-labeling after a 10 day anoxic incubation with ^{13}C-labeled sodium bicarbonate. The y-axis displays the ^{13}C-EAF value for each OTU (estimated atomic fraction%), and the error bars represent 90% confidence intervals across three biological replicates (1000 bootstrap replications). ^{13}C-labeling is considered significant if the lower range of the 90% confidence interval does not cross zero. Note that many of the OTUs with the highest amount of labeling were affiliated with the chemolithoautotrophic group Desulfobulbaceae (n=9 OTUs), which is a group of anaerobic chemolithoautotrophic cable bacteria.

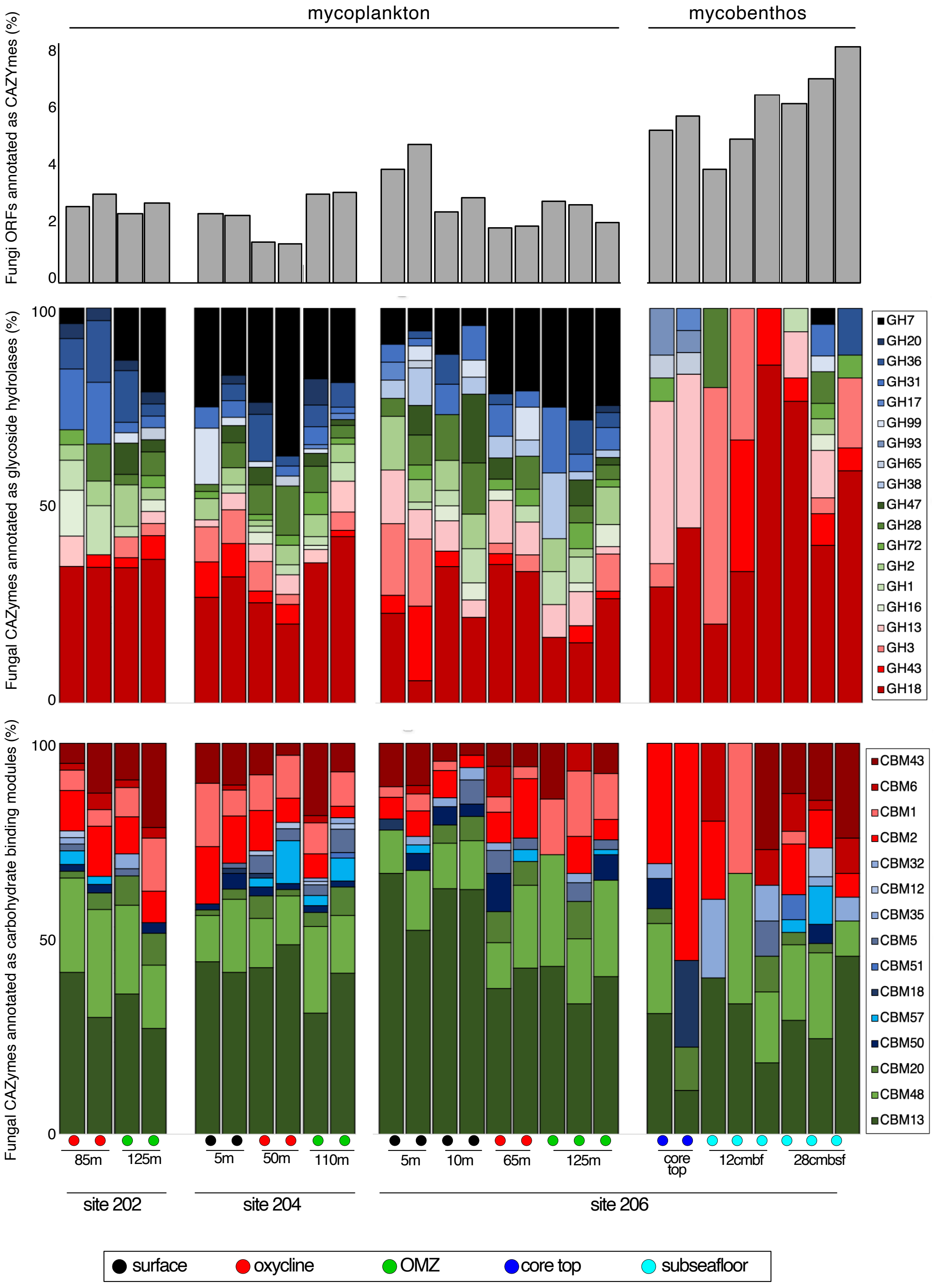

Figure S9. Top panel: Relative abundance of fungal CAZymes detected in the metatranscriptomes. Middle panel: Relative abundance of fungal carbohydrate binding modules (CBMs) in the metatranscriptomes. Bottom panel: Relative abundance of fungal glycoside hydrolases (GH) in the metatranscriptomes.
